# Pre-catalysts for the hydrogen evolution reaction: from dynamic reconstruction to rational design

**DOI:** 10.1039/d6sc05913a

**Published:** 2026-08-28

**Authors:** Xin Zhang, Jinrui Hu, Xinyi Yao, Wei Yan, Linda Zhang, Yawen Tang, Meng Li, Hao Li, Gengtao Fu

**Affiliations:** a Jiangsu Key Laboratory of Long-duration Energy Storage Battery Technology, Jiangsu Collaborative Innovation Center of Biomedical Functional Materials, Jiangsu Key Laboratory of Micro Nano Sensing and Separation Science for Analytical Chemistry, School of Chemistry and Materials Science, Nanjing Normal University Nanjing 210023 China limeng@njnu.edu.cn gengtaofu@njnu.edu.cn; b Advanced Institute for Materials Research (WPI-AIMR), Tohoku University Sendai 980-8577 Japan li.hao.b8@tohoku.ac.jp

## Abstract

The hydrogen evolution reaction (HER) is central to green hydrogen production, yet its sluggish kinetics impose a fundamental limit on energy conversion efficiency. Catalyst design is undergoing a paradigm shift from static optimization toward dynamic control, as the catalytically active phase often emerges *in situ* at reductive potentials, giving rise to the concept of the “pre-catalyst”. While oxidative reconstruction in the oxygen evolution reaction (OER) has been extensively studied, pre-catalyst reductive reconstruction under HER conditions involves metal–hydrogen (M–H) chemistry and remains mechanistically elusive. This review presents a “dynamic reconstruction to rational design” framework for HER pre-catalysts, surveying reconstruction pathways, dissecting extrinsic-intrinsic driving forces (potential, pH *vs.* electronic structure, and defects), and distilling strategies (defect engineering, chemical state modulation, *etc.*) that pre-encode the target active phase. We further highlight the methodological synergy of multimodal *operando* characterization and multiscale computation and argue that HER reconstruction is a thermodynamically driven, chemically pre-programmable evolutionary process rather than an incidental phenomenon. This reconstruction-centered view offers a design logic that extends beyond the HER to other reductive electrocatalytic systems.

## Abstract 2

This review presents a “dynamic reconstruction to rational design” framework of pre-catalysts towards the hydrogen evolution reaction.

## Graphical abstract

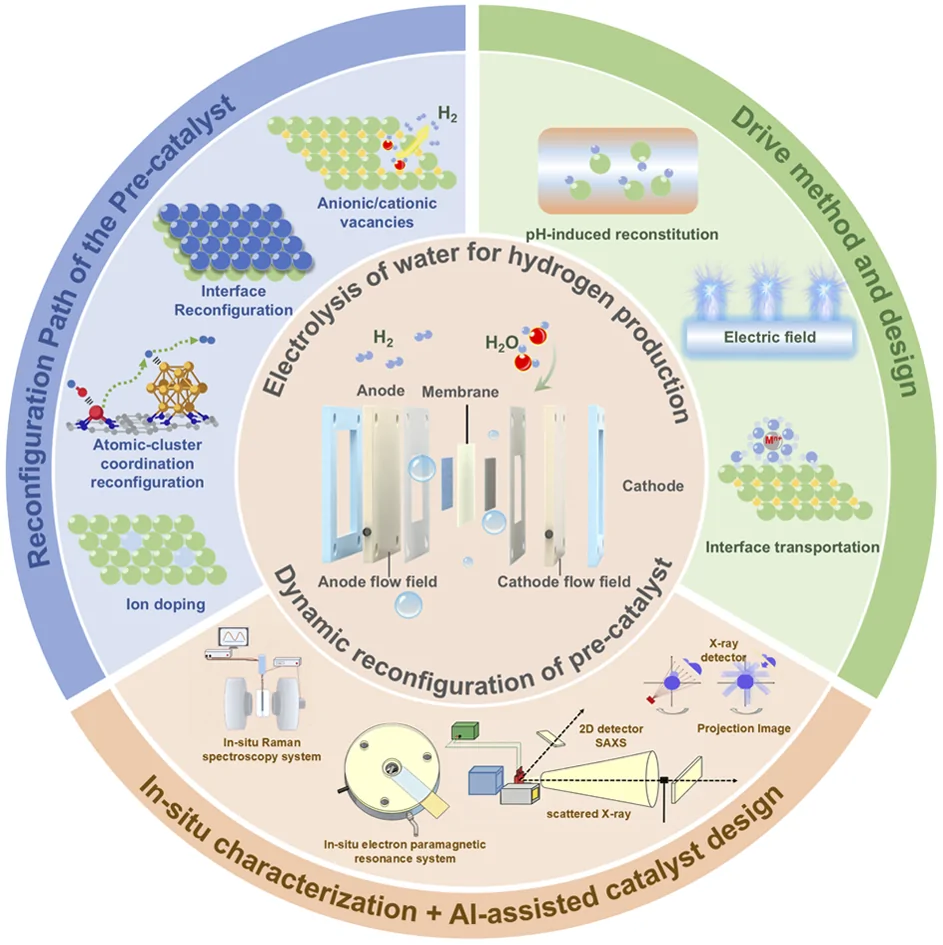

## 1 Introduction

Green hydrogen production *via* water electrolysis is central to decarbonizing energy systems, yet its efficiency is limited by the cathodic hydrogen evolution reaction (HER).^1–5^ The HER serves as the common cathodic half-reaction across all major water electrolysis technologies, including alkaline water electrolysis (AWE), proton exchange membrane water electrolysis (PEMWE), and anion exchange membrane water electrolysis (AEMWE).^6–10^ Its intrinsic kinetics largely dictate the cell voltage and overall energy conversion efficiency. However, the sluggish kinetics of the HER, particularly in alkaline and neutral media with non-noble metal catalysts, constitute a primary bottleneck, substantially limiting efficiency and raising hydrogen production costs.^11–13^ Deciphering the HER mechanism at the atomic scale and overcoming its multi-step kinetic barriers therefore represent the key challenges for advancing green hydrogen technology.^14–17^

For decades, efficient HER electrocatalyst design has followed a straightforward premise, optimizing the composition, morphology, and crystal phase of as-synthesized materials to establish static structure–activity relationships.^18–26^ This approach inherently assumes structural invariance under operating conditions, which fails at the harsh potentials of actual electrolysis, where materials undergo significant *in situ* evolution. Recent advances in *operando* and *in situ* characterization have catalyzed a paradigm shift, moving the field from steady-state descriptions toward the precise tracking of dynamic structural evolution.^27–29^ This shift has given rise to the concept of HER “pre-catalysts”. Under cathodic polarization, these pre-catalysts are structurally transformed into a new persistent working phase/state that is distinguishable from the pristine precursor and actively participates in the HER. While the term “pre-catalyst” has long been used to describe catalyst precursors that require activation, its significance in the field of electrocatalysis was powerfully demonstrated through seminal studies on the oxygen evolution reaction (OER).^30,31^ Early and influential examples include the genuine active species for many transition metal chalcogenides and pnictides as oxide layers generated *in situ* on the surface.^32–34^ These findings established the “pre-catalyst” paradigm within the electrocatalysis community and have since inspired its extension to a wide range of systems.^35^ A particularly compelling demonstration is the work of Zuo *et al.*, in which Fe–Ni-based pre-catalysts transfer “structural disorder” to the *in situ*-formed oxyhydroxide, correlating positively with enhanced OER activity.^36^ This finding highlights the possibility that the structural features of the final active phase can be programmed by pre-designing the initial catalyst.

Despite these advances in the OER, the study of HER pre-catalyst reconstruction has lagged considerably. This lag is not merely a deficit in experimental effort, but rather reflects a fundamental difference in chemical driving forces. OER reconstruction occurs at oxidative potentials and is driven by high-valent metal-oxo formation.^37,38^ HER reconstruction, in contrast, proceeds under strongly reducing conditions and introduces the unique dimension of metal–hydrogen (M–H) chemistry, encompassing hydrogen adsorption and desorption, lattice insertion, hydride formation, and hydrogen spillover.^39–41^ These processes are not encountered in the oxidative reconstruction of the OER and are notoriously difficult to track *in situ*. In alkaline HER, the additional H–OH bond cleavage step introduces further mechanistic complexity. This often makes water dissociation kinetics more rate-determining than hydrogen binding itself.^42,43^ Despite these mechanistic complexities, progress has been made in unraveling the HER mechanism and translating this understanding into advanced catalyst design. The classical single-descriptor framework based on hydrogen binding energy (Δ*G*_H^*^_) has been expanded into a multidimensional kinetic network that includes water adsorption and dissociation, *OH desorption, hydrogen spillover, and interfacial water structure.^44–46^ This enriched understanding has catalyzed a range of advanced design strategies, ranging from heterointerface engineering and orbital coupling to multi-site synergy and interfacial water network reconfiguration.^47–54^ Collectively, these approaches aim not only to optimize a single active site, but rather to encode multi-step kinetics and dynamic interfacial behavior into the catalyst design. These advances convey a unifying message. The catalytically active species in HER pre-catalysts is therefore not the pre-existing structure, but the one dynamically generated at the interface during electrolysis. Yet the majority of current studies still treat reconstruction as a post explanation of performance, rather than as a design variable to be pre-programmed into the initial catalyst. Compared with the mature OER pre-catalyst design framework, HER reconstruction research remains fragmented and lacks a coherent guiding logic.^55–59^

**Fig. 1:**
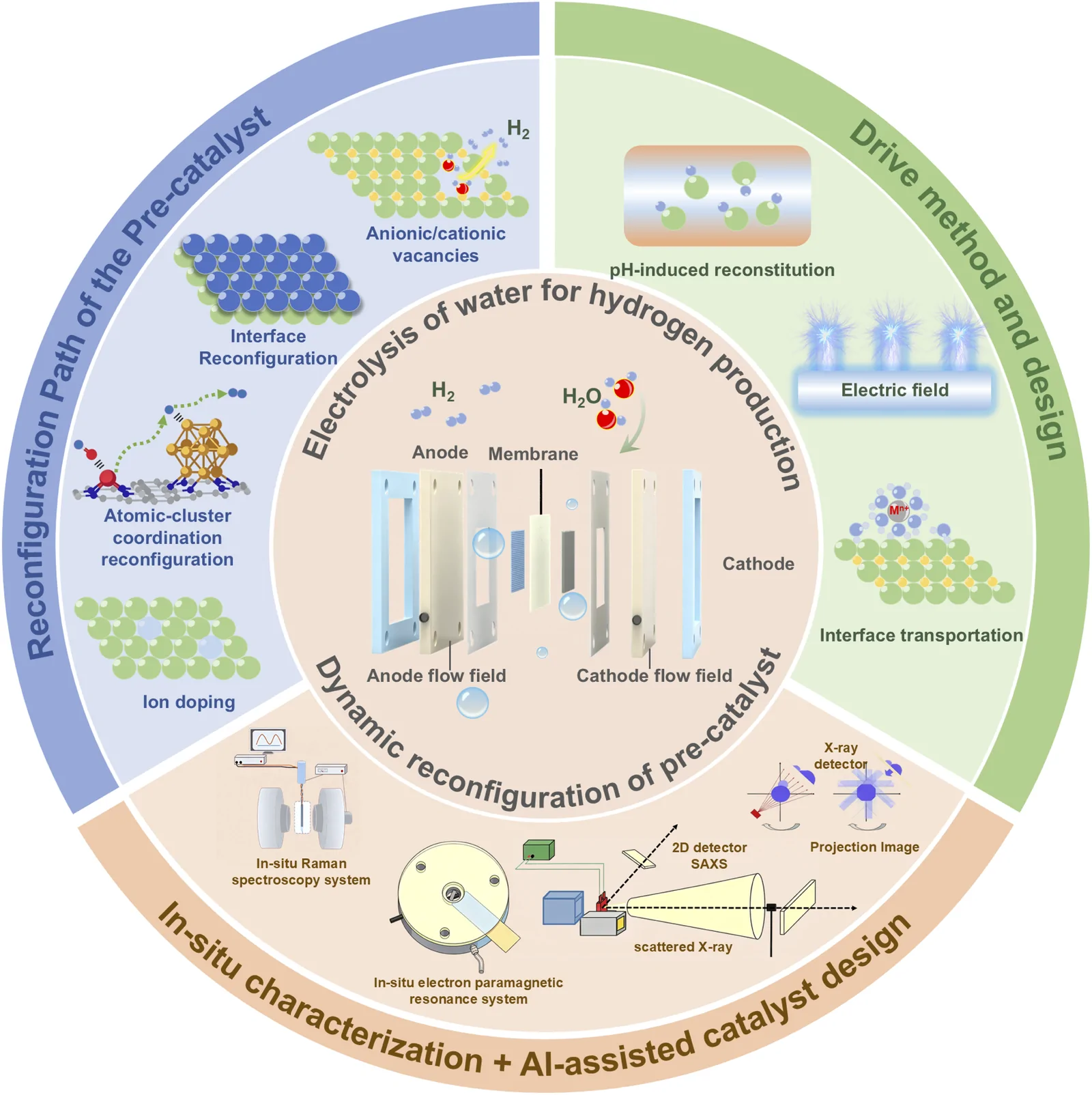
Schematic overview of the research framework for dynamic reconstruction and rational design of HER pre-catalysts.

Given the fragmented nature of current HER reconstruction studies and the lack of a systematic design logic, a comprehensive review that consolidates these disparate findings into a unified framework is urgently needed. The central objective of this review is to establish a holistic “dynamic reconstruction to rational design” paradigm specifically for HER pre-catalysts (Fig. 1). To this end, we first survey the reconstruction pathways and mechanisms across representative material classes, revealing both the commonalities and distinctive features that emerge under reducing conditions. We then dissect the intrinsic and extrinsic driving forces that govern these reconstruction behaviors and distill the corresponding rational design strategies. Finally, we integrate multiscale *operando* characterization with computational modeling to demonstrate how these dynamic processes can be resolved and mechanistically understood, while highlighting the challenges toward predictive reconstruction design.

## 2 Reconstruction pathways and mechanisms of HER pre-catalysts

Understanding the chemical driving forces that govern dynamic reconstruction is the essential prerequisite for rational pre-catalyst design. Reconstruction is a dynamic structural response process that occurs at bias potential. The HER “pre-catalyst” is defined as the initial precursor that undergoes irreversible structural reconstruction under cathodic polarization to generate a persistent working phase that directly participates in the HER. In contrast to the surface oxidative reconstruction driven by high anodic potentials in the OER, the HER reconstruction occurs under strongly reducing conditions, where pre-catalysts undergo structural evolution toward catalytically relevant working states in response to changes in chemical potential.

**Fig. 2:**
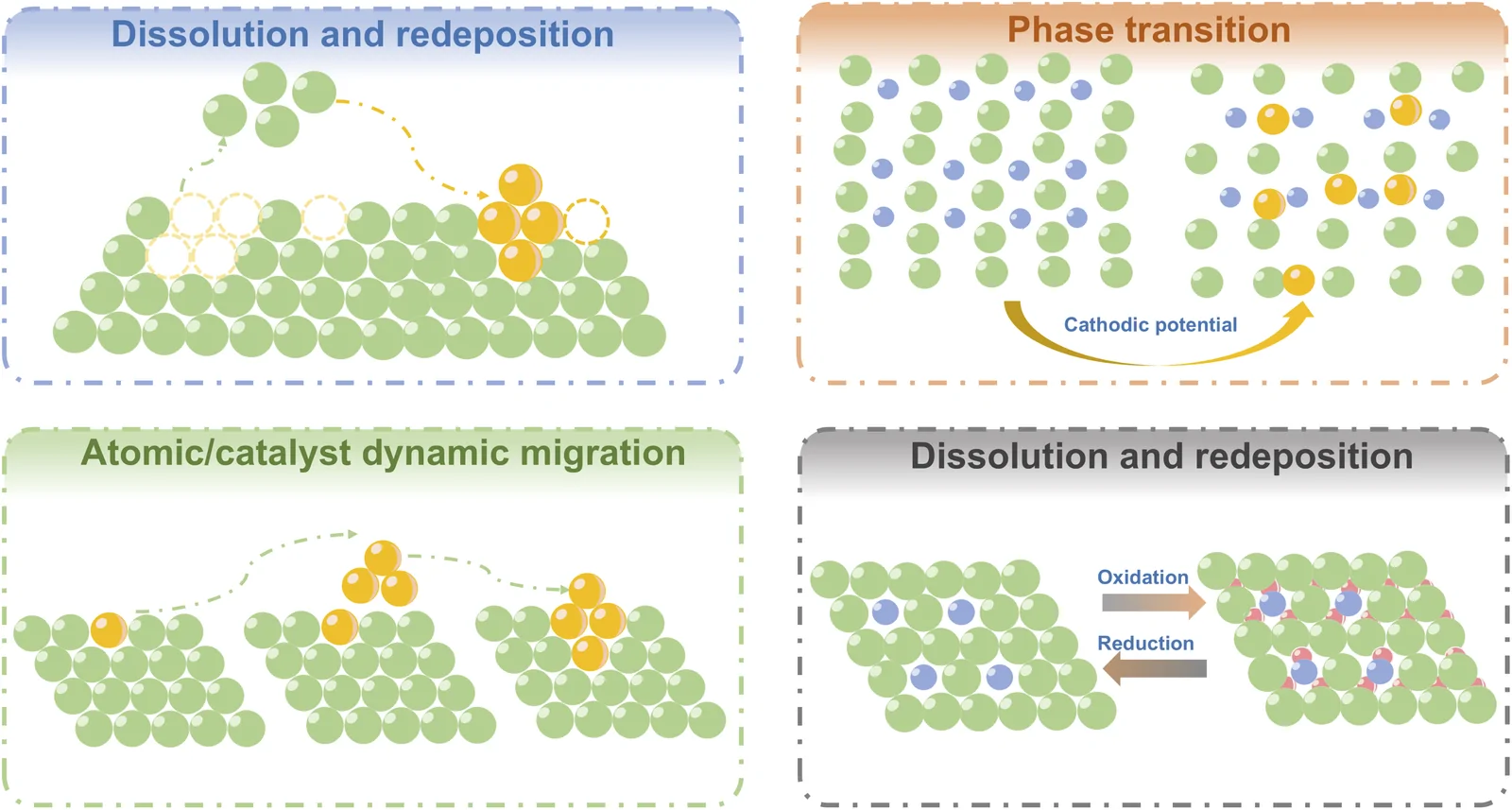
Schematic illustration of the four representative reconstruction pathways of HER pre-catalysts driven by intrinsic materials chemistry.

The reconstruction process of HER catalysts yields three distinct categories: (i) reconstruction converges to a stable, active working phase with sustained HER performance; (ii) excessive reconstruction leads to structural collapse, severe dissolution, particle coarsening, or active-phase detachment, resulting in net loss of active sites and catalytic degradation; and (iii) reconstruction reaches a metastable or intermediate state with moderate or transient activity but gradually deactivates under prolonged operation. It should be emphasized that reconstruction itself does not guarantee an increase in activity. The final state of HER catalysts can either converge to a new active state or may lead to the collapse of the framework or passivation due to excessive corrosion, resulting in irreversible degradation of performance.^60^ Pre-catalyst reconstruction is not uncontrolled collapse but results from the interplay between intrinsic evolutionary propensity and the electrochemical environment. Its distinction from degradation hinges on evaluating active-site accessibility, dissolution extent, electrochemical stability, and the performance-structure correlation of the reconstructed state. Also, such structural evolution should be distinguished from reversible surface responses that recover upon removal of the applied potential, such as adsorption/desorption, ligand exchange, surface charging, or double-layer reorganization.^61–63^ The reconstruction behavior of a pre-catalyst is fundamentally determined by its intrinsic materials properties, including chemical composition, crystal structure, and atomic architecture.^64–66^ These properties dictate the thermodynamic driving forces, the kinetic pathways available for structural evolution, and ultimately the nature of the final active phase. To capture the diversity arising from different materials properties, this chapter surveys four representative classes of HER pre-catalysts with distinct structural and compositional characteristics (Fig. 2). Comparing these classes shows how the starting material chemistry dictates the reconstruction pathway and ultimately the catalytic activity.

### 2.1 Metal chalcogenides and phosphides

The reconstruction of metal chalcogenides and phosphides is typically triggered by the selective leaching of anions at cathodic potentials.^13,67–69^ The dissolution of elements such as S, Se, or P from the lattice generates vacancies that serve a dual function.^70^ They create coordinatively unsaturated metal centers with potentially high intrinsic activity, and more importantly, they often induce a complete phase transformation of the surface layer, generating metal hydroxide or oxy(hydroxide) overlayers *in situ*.^62,71^ The initial anion identity and coordination geometry thus preset both vacancy population and the electronic character of the final active center.

**Fig. 3:**
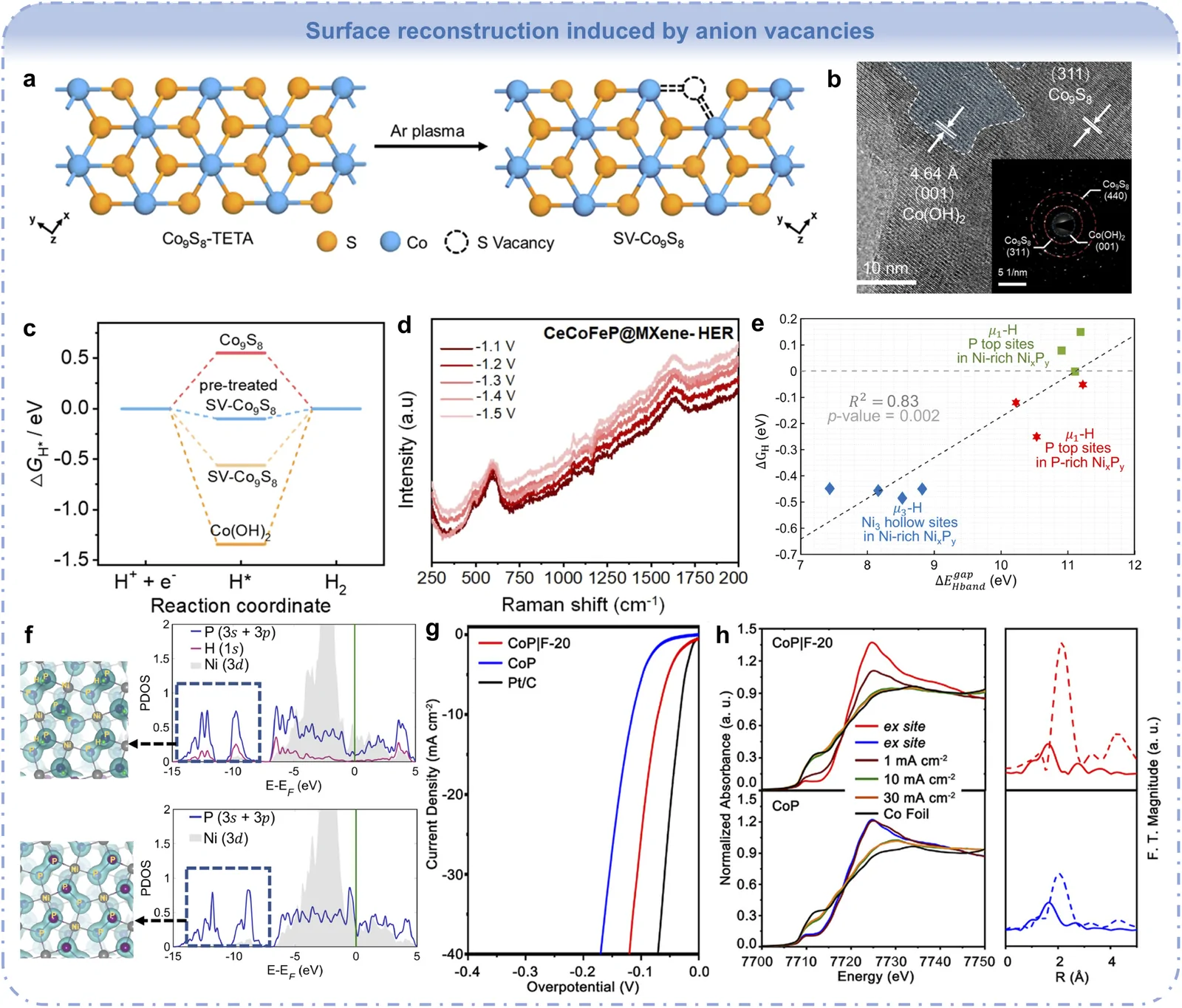
(a) Schematic illustration of sulfur vacancy creation in Co_9_S_8_*via* Ar plasma treatment. (b) HRTEM image of the Co(OH)_2_/SV-Co_9_S_8_ heterointerface. (c) Free energy diagrams for the HER on various surfaces.^72^ Copyright 2022, American Chemical Society. (d) *In situ* Raman spectra of CeCoFeP@MXene during the HER.^71^ Copyright 2025, Elsevier. (e) Correlation between Δ*E*^gap^_Hband_ and Δ*G*_H^*^_ for different Ni_*x*_P_*y*_ sites. (f) PDOS and ILDOS analyses of P–P and Ni_3_ sites.^74^ Copyright 2023, American Chemical Society. (g) HER polarization curves of CoP|F-20, CoP, and commercial Pt/C in 0.5 M H_2_SO_4_. (h) *Operando* Co K-edge EXAFS spectra of CoP|F at different current densities.^75^ Copyright 2025, Wiley-VCH.

The catalytic performance of the reconstructed chalcogenides/phosphides therefore hinges on the synergistic interplay between the residual vacancies and the newly formed phases. This vacancy-triggered reconstruction is exemplified by the work of Wu *et al.* on Co_9_S_8_ nanosheets with abundant sulfur vacancies.^72^Fig. 3a schematically illustrates the process of introducing sulfur vacancies *via* Ar plasma treatment. The vacancies do not serve as permanent active sites but rather as a switch that triggers the *in situ* formation of a Co(OH)_2_/SV-Co_9_S_8_ heterointerface. HRTEM confirms the *in situ* formation of Co(OH)_2_ and its construction of a heterointerface with Co_9_S_8_ during alkaline HER (Fig. 3b). The final heterostructure, through synergy between Co(OH)_2_ and residual vacancies, achieves a near-ideal Δ*G*_H^*^_ of −0.10 eV (Fig. 3c), whereas the vacancy-containing intermediate binds hydrogen too strongly. These results associate the reconstructed Co(OH)_2_/SV-Co_9_S_8_ heterointerface with the enhanced HER activity and support its catalytic relevance. Also, the generality of anion-leaching reconstruction can be extended from sulfides to phosphides. Pan *et al.* observed that MXene-supported CeCoFe phosphides undergo significant phosphorus leaching under alkaline HER conditions, generating phosphate-modified metal hydroxide or oxyhydroxide species as HER active sites.^71^ The appearance of M–OH and P–O peaks in *in situ* Raman spectroscopy and the observation of the shift of M–P peak also confirmed this phenomenon (Fig. 3d). This finding establishes that anion-leaching-driven reconstruction is a general phenomenon across different classes of chalcogenide and phosphide materials.

A further question concerns whether the reconstructed phase can be optimized by pre-catalyst design. Kim *et al.* showed that sulfur introduced into NiFeOOH is not completely leached out, but persists within the active phase, increasing electron density on Ni and Fe sites and optimizing hydrogen adsorption.^73^ Thus, heteroatom doping can serve not only as a precursor for generating the active phase but also as an electronic modulator of the final active phase. Banerjee *et al.* provided a theoretical framework for understanding the vacancy-activity relationship. DFT calculations reveal that alkaline conditions drive the formation of phosphorus vacancies in nickel phosphides, exposing Ni_3_ hollow sites and residual P–P dimer sites.^74^ As shown in the correlation between the covalent hybridization strength descriptor Δ*E*^gap^_Hband_ and Δ*G*_H^*^_ in Fig. 3e, P–P sites exhibit near-thermoneutral adsorption, while Ni_3_ sites bind hydrogen too strongly. Fig. 3f further presents projected density of states (PDOS) and integrated local density of states (ILDOS) analyses, demonstrating that surface P–P bonds provide strong directional covalent hybridization with H 1s, thereby explaining the activation mechanism of P–P sites from the electronic structure origin. This “vacancy poisoning *versus* P–P activation” model establishes that HER performance is governed by the dynamic equilibrium between vacancies and specific surface sites, rather than by vacancy quantity alone. The mechanistic insights from these reconstruction studies are not limited to alkaline media. A recent study by Wu *et al.* demonstrated that fluorine-modified CoP (CoP|F) shows efficient acidic HER performance and stability in a proton exchange membrane water electrolyzer (Fig. 3g).^75^*In situ* X-ray absorption spectroscopy (Fig. 3h) reveals that fluorine incorporation promotes the cleavage of Co–P bonds, leading to the formation of an amorphous metallic Co phase with enhanced HER activity. Density functional theory calculations further show that the presence of fluorine in the CoP_1−*x*_ lattice facilitates the formation of phosphorus vacancies under HER conditions, generating more zero-valent cobalt active sites. This reconstructed catalyst achieved performance comparable to that of commercial Pt/C in a 38 cm^2^ PEM electrolyzer. This finding extends pre-catalyst design beyond alkaline systems, showing that non-noble metals can be activated even under acidic PEM conditions.

### 2.2 Alloys

In contrast to the selective anion leaching observed in chalcogenides and phosphides, reconstruction in alloy systems predominantly takes the form of dealloying and elemental segregation.^76–78^ This involves the selective dissolution of thermodynamically less stable components at reducing potentials, driving dynamic self-optimization of surface composition and atomic arrangement. The resulting chemically heterogeneous surfaces can generate bifunctional interfaces that synergistically catalyze multi-step reactions.^79–81^

**Fig. 4:**
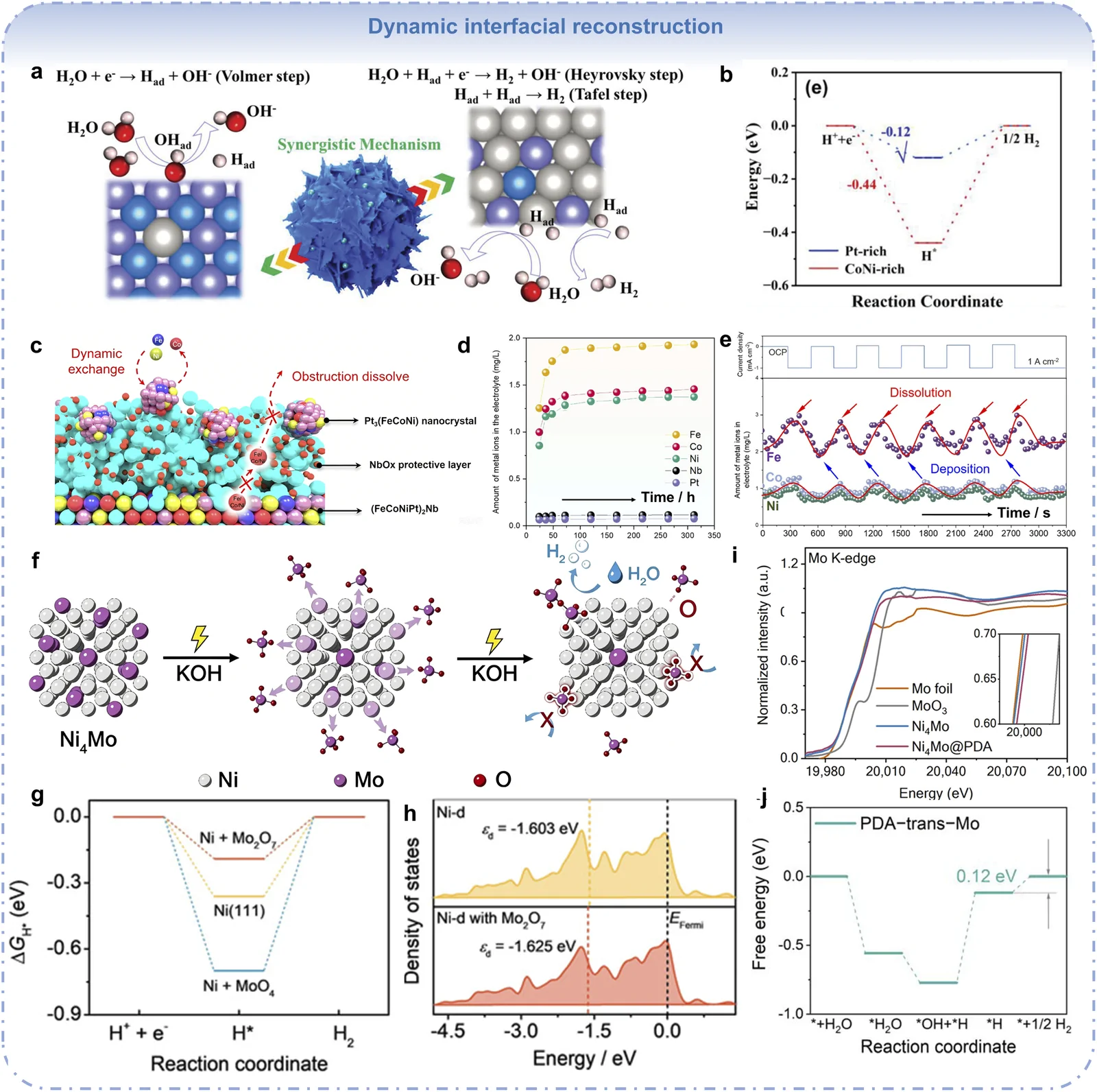
(a) Schematic illustration of the synergistic catalytic mechanism of alloy segregation in CoNiPt catalysts. (b) Calculated Δ*G*_H^*^_ values for Pt-rich and CoNi-rich phases.^83^ Copyright 2023, Wiley-VCH. (c) Schematic diagram of the dynamic equilibrium mechanism of the NP-Pt_3_ electrode. (d) Dissolution profiles of Fe, Co, and Ni elements during durability testing at alternating OCP and HER potentials. (e) Schematic diagram of the dissolution-deposition dynamic equilibrium mechanism.^84^ Copyright 2025, Wiley-VCH. (f) Schematic illustration of the behaviors of Mo in Ni_4_Mo alloy during alkaline HER. (g) Free energy diagrams for the HER. (h) DOS of Ni 3d orbitals with and without Mo_2_O_7_^2−^adsorption.^86^ Copyright 2021, Wiley-VCH. (i) FT-EXAFS fitting curves of Ni_4_Mo@PDA at Ni and Mo K-edges. (j) Optimized configurations and corresponding Gibbs free energy profiles for the HER on the PDA-trans-Mo site.^87^ Copyright 2026, American Chemical Society.

Recent advances in Pt-based HER electrocatalysts have been systematically reviewed by Guo *et al.*, with particular emphasis on synthetic strategies for reducing Pt loading while maintaining high catalytic activity through alloying and interfacial electronic modulation.^82^ An emerging dimension in Pt-alloy catalyst design is the dynamic surface reconstruction that occurs under operating conditions. This dealloying mechanism is well illustrated in the work of Pan *et al.* on carbon-coated CoNiPt alloys.^83^ In the acid HER process, the alloy selectively leached out unstable Co and Ni atoms, generating a Pt-enriched surface layer and a CoNi-enriched core. This chemically induced dealloying mimics the selective dissolution that would occur at reducing potentials. The schematic of the synergistic catalytic mechanism of alloy segregation (Fig. 4a) systematically summarizes how this phase-separated structure enables bifunctional synergistic catalysis. Meanwhile, DFT calculations (Fig. 4b) reveal that the Pt-enriched phase exhibits near-ideal hydrogen adsorption free energy (Δ*G*_H^*^_ = −0.12 eV), while the CoNi-enriched core, with stronger OH* binding capability, facilitates water dissociation. This phase-separated structure enables the material to deliver performance exceeding that of commercial Pt/C at very low Pt loading.

The reconstruction of alloy catalysts becomes even more dynamic under strongly reducing and acidic conditions. Li *et al.* reported a dissolution-deposition equilibrium mechanism in a (FeCoNiPt)_2_Nb porous high-entropy alloy for acidic PEMWE (Fig. 4c).^84^ During HER operation, *in situ* ICP-MS reveals that Fe, Co, and Ni dissolved from the surface into the electrolyte at open-circuit potential. Element-specific dissolution profiles during durability testing reveal the gradual establishment of a dynamic equilibrium. The periodic fluctuation of ion concentrations at alternating OCP and HER potentials (Fig. 4d) further confirms that the rates of dissolution and deposition are comparable. As schematically depicted in Fig. 4e, the NbO_*x*_ layer and high-entropy effect jointly suppress dissolution, while multicomponent alloying promotes deposition, together maintaining a self-renewing surface. In cyclic potential switching, the ion concentrations fluctuated within a narrow range and maintained a dynamic equilibrium, demonstrating that the dissolution and deposition rates were comparable. The synergistic regulation of dissolution and deposition rates established a dynamic equilibrium, enabling the catalyst surface to continuously self-renew while retaining both high activity and stability.^85^

While the acid HER environment represents an *in situ* approach to dealloying, the reconstruction of alloys can also occur under alkaline electrochemical conditions. A distinct type of reconstruction behavior was unveiled by Du *et al.* in the Ni_4_Mo alloy system.^86^ Contrary to the conventional assumption that metallic alloys remain structurally invariant under alkaline cathodic conditions, this work revealed that during alkaline HER, Mo in Ni_4_Mo is not stable. Under cathodic polarization, metallic Mo in Ni_4_Mo is oxidized and dissolves from the alloy surface in the form of MoO_4_^2−^(Fig. 4f). *In situ* Raman spectroscopy further reveals that a portion of the dissolved MoO_4_^2−^ adsorbed on the electrode surface undergoes polymerization to form Mo_2_O_7_^2−^. This dissolution-polymerization process dynamically modifies the surface chemical environment. Theoretical calculations (Fig. 4g) demonstrate that the adsorption of Mo_2_O_7_^2−^ on the electrode surface lowers the desorption barrier of adsorbed hydrogen, confirming the promotional role of polymerized molybdate in the HER. Density of states analysis (Fig. 4h) further provides an electronic-structure explanation, revealing a downward shift of the Ni 3d d-band center that facilitates hydrogen desorption.

The electrochemical reconstruction of alloys can be further modulated through surface modification. Kong *et al.* developed a polydopamine (PDA) nanocage coating on Ni_4_Mo electrocatalysts through a programmable electrochemical assembly method.^87^ The catechol groups of PDA form strong coordination bonds with Ni and Mo sites on the alloy surface, as evidenced by EXAFS coordination peaks (Fig. 4i). This interfacial interaction suppresses the continuous dissolution of Mo from Ni_4_Mo under harsh electrochemical conditions, which would otherwise lead to phase transformation and loss of active sites. First-principles calculations reveal that PDA-mediated Ni and Mo coordination constructs a multi-site catalytic network that modulates the interfacial energetics of HER intermediates, thereby accelerating HER kinetics, with the H adsorption free energy at PDA-trans-Mo sites reaching only 0.12 eV, close to the thermoneutral value (Fig. 4j). The porous rigid-flexible nanocage architecture not only stabilizes the alloy against dissolution but also promotes electrolyte penetration and rapid gas evolution. In this case, surface modification deliberately guides the reconstruction pathway by suppressing detrimental dissolution while preserving a favorable surface chemical environment.

Alloy reconstruction pathways originate from the driving force of lowering the surface free energy under cathodic bias. This spontaneous process generates chemically differentiated surfaces that spatially decouple elementary steps, which single-phase materials can hardly achieve.^88,89^ The dynamic nature of this evolution also provides a pathway for continuous surface self-renewal under operating conditions. The key to rational pre-catalyst design lies in tuning the initial alloy composition and structure to direct the reconstruction pathway toward the desired functional interface.

### 2.3 Single-atom and cluster catalysts

Single-atom catalytic sites and clusters have emerged as a new frontier in catalysis and materials science.^90,91^ When the size of active centers is reduced to single atoms or sub-nanometer clusters, their reconstruction behavior under electrochemical conditions shifts from the evolution of long-range ordered structures to the dynamic modulation of local coordination environments and electronic states.^92–94^ At reducing potentials, the coordination shell of individual metal atoms may undergo distortion or reconstruction, while isolated metal sites and adjacent atomic clusters can generate strong synergistic effects through electron transfer or intermediate spillover. If the reconstruction of chalcogenides and alloys can be described as a wholesale bulk phase transformation, then that of single atoms and clusters is a more delicate fine-tuning of electronic and coordination structures. Such atomic-scale dynamic interactions enable catalysts to circumvent the constraints of conventional linear scaling relations.

**Fig. 5:**
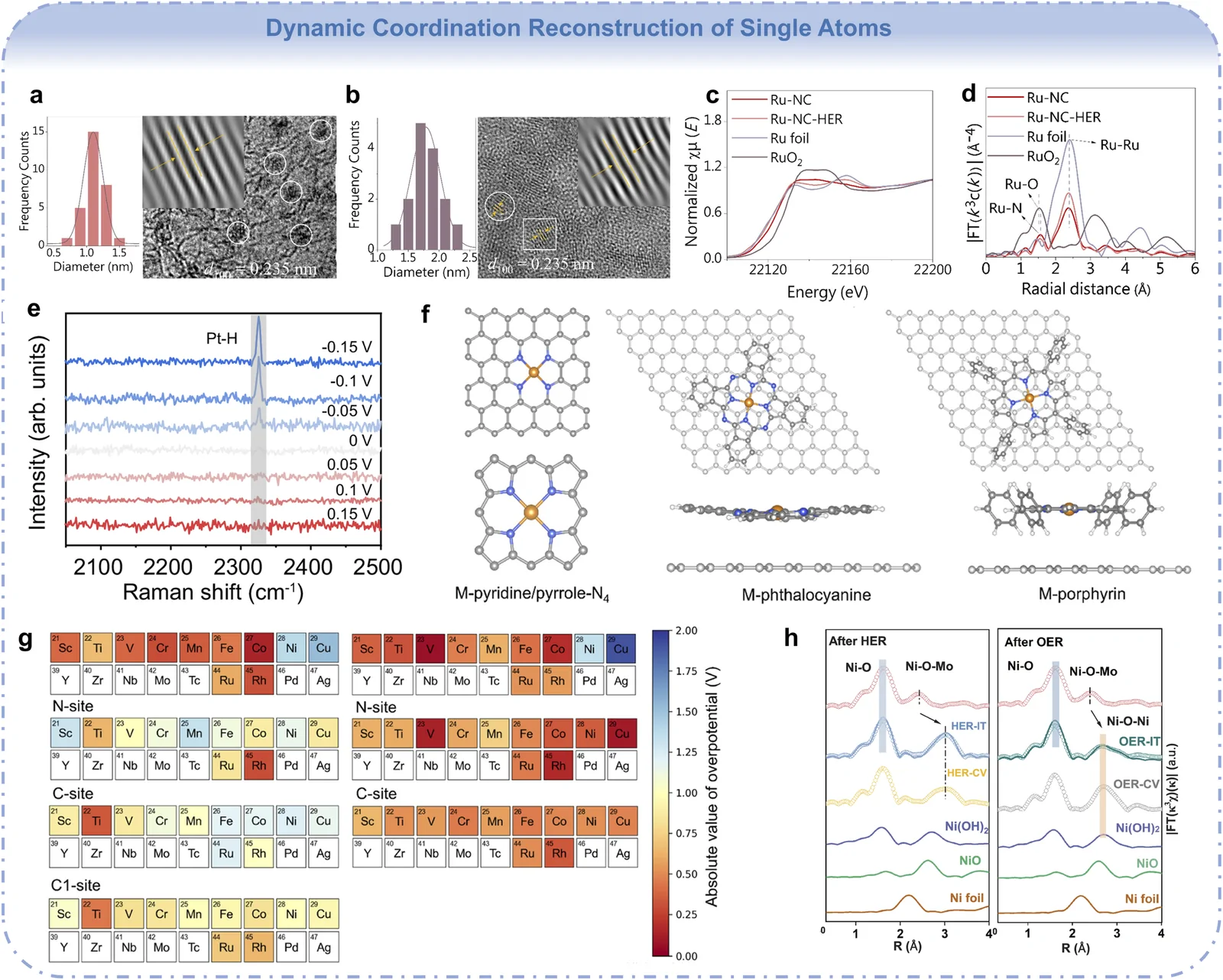
(a) HRTEM images and (b) particle size distribution diagrams of Ru-NC and Ru-NC-HER. (c) Ru K-edge XANES spectra. (d) Fourier transforms of the Ru K-edge EXAFS spectra.^95^ Copyright 2026, American Chemical Society. (e) *In situ* Raman spectra of NCNT-Ni/Pt at different potentials.^97^ Copyright 2026, Springer Nature. (f) Atomistic models of pyridinic M-N_4_, pyrrolic M-N_4_, MPc, and MPor structures. (g) Theoretical HER overpotentials at different adsorption sites.^98^ Copyright 2025, Wiley-VCH. (h) *Ex situ* XAS spectra of Ni K-edge of NiSA-O/Mo_2_C after the HER and OER.^99^ Copyright 2024, Springer Nature.

The most direct manifestation of reconstruction in atomically dispersed catalysts is the migration and coalescence of metal species under operating conditions. Du *et al.* investigated an atomically dispersed Ru catalyst on nitrogen-doped carbon (Ru/NC) during alkaline HER.^95^ After the HER, Ru single atoms nearly disappeared while Ru clusters grew from 1.10 nm to 1.80 nm (Fig. 5a and b), accompanied by a weakened Ru–N coordination and an intensified Ru–Ru metallic bond (Fig. 5c and d). A similar phenomenon was observed by Wang *et al.* in atomically dispersed Pt catalysts, where cathodic potentials weakened Pt–N bonding and induced partial Pt single atoms to dynamically aggregate into small clusters.^96^ These observations point out that atomic dispersion is not necessarily the thermodynamic endpoint under HER conditions, and the dynamic formation of clusters or nanoparticles can serve as the catalytically active sites for the HER.

Beyond atomic migration and support evolution, reconstruction can also occur through more subtle pathways. Long *et al.* reported a Pt single-site catalyst (NCNT-Ni/Pt) with an initial N_2_–Pt–Cl_2_ coordination configuration.^97^ During HER operation, the dynamic evolution of Pt–Cl bonds regulated the catalytic performance, with Cl ligands progressively replaced by H to form active centers (Fig. 5e). Ye *et al.* systematically investigated this phenomenon in M–N–C single-atom catalysts by constructing 76 computational models spanning 3d, 4d, and 5d transition metals, including pyridinic M-N_4_, pyrrolic M-N_4_, metal phthalocyanine, and metal porphyrin configurations (Fig. 5f).^98^ Upon hydrogen adsorption, the H atom pulls the metal center out of the graphene plane, inducing a pronounced protrusion. Importantly, this M-N_4_ coordination reconstruction is retained even after H* desorption, meaning that the clean-surface state of the catalyst after the reaction is structurally distinct from its as-synthesized form. For metals with weak hydrogen adsorption such as Ni and Cu, the N atoms originally serving as the coordination environment can themselves become the active centers (Fig. 5g). These cases demonstrate that coordination dynamics, occurring without extensive atomic migration, can serve as the primary mode of reconstruction, where the geometric and electronic structure of a catalytic center co-varies with reactants and intermediates.

The reconstruction behavior of single-atom catalysts also exhibits a strong dependence on the applied potential. Hou *et al.* reported Ni single atoms anchored on oxygen-incorporated Mo_2_C *via* Ni–O–Mo bridge bonds (Ni_SA_-O/Mo_2_C).^99^ Quasi *in situ* synchrotron X-ray absorption spectroscopy reveals that after the HER, the Ni species remained atomically dispersed but with altered coordination numbers and bond lengths; after the OER, by contrast, the atomically dispersed Ni agglomerated into small clusters (Fig. 5h). This potential-dependent divergence underscores that the same single-atom catalyst can undergo distinctly different structural evolutions depending on the reaction environment, highlighting the necessity of *operando* characterization for identifying catalytically active species under specific operating conditions. Zhu *et al.* reported a Ru catalyst supported on layered sodium cobaltate (NaCoO_2_, NC) that contained both Ru single atoms and adjacent amorphous Ru hydroxide clusters.^100^ At HER cathodic potentials, the Ru hydroxide clusters underwent irreversible reduction to metallic Ru, and this metallic state was preserved even after returning to open-circuit voltage, with only minor surface reoxidation. These observations collectively illustrate that the reconstruction pathway of single-atom catalysts is not only species-dependent but also highly sensitive to the applied potential. The same catalyst may remain atomically dispersed under one condition yet aggregate into clusters under another, or undergo irreversible reduction-driven transformation that persists even after the potential is removed.

Unlike chalcogenides and alloys, single-atom catalysts reconstruct locally at the coordination level. The reconstruction behaviors discussed above do not represent degradation but an intrinsic feature of their high surface energy. The high surface free energy and metastable coordination of atomically dispersed species render them intrinsically susceptible to electrochemical driving forces, and their evolution under operating conditions is not a degradation pathway but an integral feature of their catalytic function. This recognition shifts the design paradigm for single-atom catalysts from stabilizing a fixed configuration toward encoding a favorable evolutionary trajectory into the initial coordination environment.

### 2.4 Interfacial architecture catalysts

**Fig. 6:**
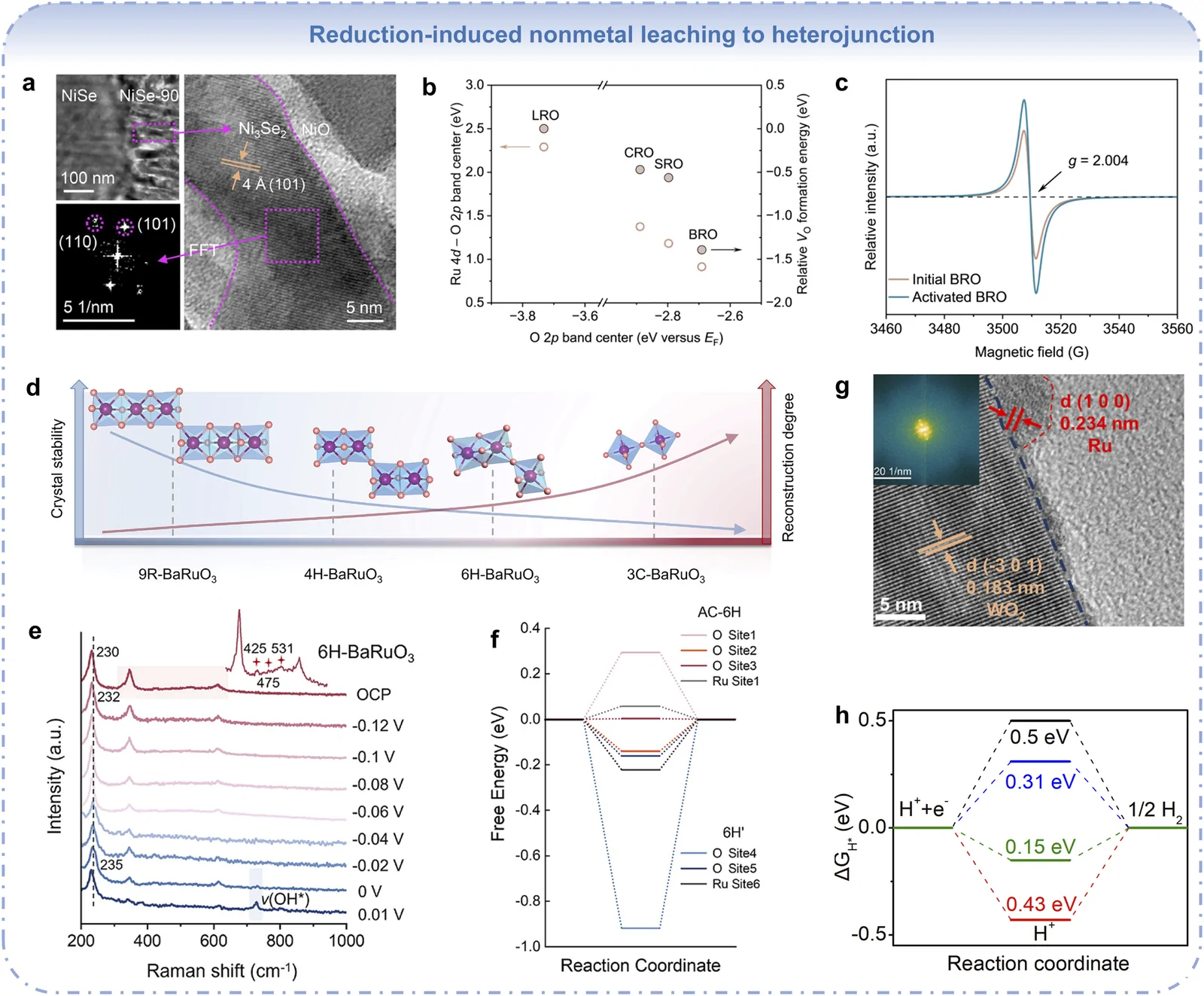
(a) Schematic illustration of the multi-step cascade reconstruction of NiSe into the Ni_3_Se_2_/NiO heterointerface during alkaline HER.^105^ Copyright 2026, Wiley-VCH. (b) Diagram of the difference between the Ru 4d band center and the O 2p band center (left) and the O vacancy formation energy relative to LRO (right) *versus* the O 2p band center relative to the Fermi level for ARuO_3_ perovskites. (c) EPR spectra of initial and activated BRO.^106^ Copyright 2026, American Chemical Society. (d) Schematic illustration of RuO_6_ octahedral connectivity modes in BaRuO_3_ polymorphs. (e) *In situ* Raman spectra of BaRuO_3_ polymorphs during the HER. (f) DFT-calculated Gibbs free energy diagrams for H_2_O dissociation on AC-6H and 6H′.^114^ Copyright 2026, Wiley-VCH. (g) TEM images of RWO. (h) DFT-calculated free energy diagram of hydrogen spillover on different sites.^112^ Copyright 2026, Wiley-VCH.

The preceding discussions on chalcogenides and phosphides, alloys, and single-atom catalysts share a common thread that highly active HER catalytic sites often emerge as dynamic interfaces formed under operating conditions. This observation raises the question of whether the reconstruction pathway can be deliberately guided by pre-designing the initial structure. Researchers have begun to address this question by extending the design scale from atomic-level active centers to interfacial architectures.^101,102^ The core idea is to exploit pre-constructed built-in electric fields, strain fields, or confinement effects to directionally influence component dissolution, segregation, and phase transformation, thereby programming the chemical characteristics of the final active interface.^103,104^ Ding *et al.* on NiX (X = S, Se) pre-catalysts provided clear evidence for this concept.^105^ Under cathodic polarization, NiSe undergoes a multi-step cascade reconstruction, which first transforms into an intermediate Ni_3_Se_2_ phase, followed by surface anion (Se^2−^) leaching and substitution by O species, ultimately *in situ* constructing a Ni_3_Se_2_/NiO heterointerface (Fig. 6a). The initial phase of NiSe_2_ transforms into an intermediate Ni_3_X_2_ phase, followed by gradual surface anion (S^2−^/Se^2−^) leaching and replacement by O species, ultimately *in situ* constructing the Ni_3_X_2_/Nio_2−*x*_ heterointerface. By constructing this interface, water dissociation in the Volmer step and hydrogen intermediate adsorption/recombination become spontaneously spatially decoupled. This finding indicates that the initial interfacial architecture influences the subsequent reconstruction pathway and therefore provides a basis for exploring reconstruction-guided precursor design.

A conceptually straightforward approach to constructing active heterointerfaces relies on the *in situ* exsolution of metal species from the pre-catalyst. The reconfiguration behavior can be effectively controlled by modifying the energy band structure of the pre-catalyst. Zhao *et al.* demonstrated this strategy using ARuO_3_ (A = La, Ca, Sr, Ba) perovskites as a model system.^106^ Under HER conditions, the perovskite undergoes electrochemical reduction, inducing the exsolution of metallic Ru from the lattice while generating oxygen vacancies in the oxide framework. Among ARuO_3_ perovskites, those with higher O 2p band positions (*e.g.* BaRuO_3_) exhibit lower oxygen vacancy formation energies and more pronounced Ru exsolution, which shows the most extensive surface reconstruction and HER activation (Fig. 6b). Rather than relying on dissolution from a separate phase, the active metal species are hosted within the oxide lattice and are released through electrochemical reduction, resulting in intimate contact between the exsolved metal and the oxide support (Fig. 6c). While exsolution relies on the composition-dependent electronic structure, the same perovskite framework also allows crystallographic control over the reconstruction pathway. Sun *et al.* investigated BaRuO_3_ polymorphs with different RuO_6_ octahedral connection modes, including corner-sharing and face-sharing configurations (Fig. 6d). The local connectivity of the oxide framework dictates the extent of Ba/Ru leaching and the nature of the reconstructed amorphous overlayer. Polymorphs with higher proportions of corner-sharing octahedra exhibit distinct surface reconstruction behaviors under HER conditions to form Ru_*x*_O_*y*_/6H-BaRuO_3_. *In situ* Raman spectroscopy and theoretical calculations revealed that the octahedral stacking mode influences lattice stability and metal–oxygen bond strength, thereby presetting both the thermodynamic driving force for reconstruction and the structural characteristics of the reconstructed interfacial layer (Fig. 6e and f).

The strategies discussed above rely on surface-confined reconstruction, where structural evolution occurs primarily at the exterior of the catalyst particles. Nie *et al.* demonstrated that reconstruction can be deliberately engineered to occur throughout the bulk of the material through the introduction of a soluble dopant.^107^ In a Mo-doped NiP pre-catalyst, Mo dissolves preferentially under cathodic polarization, creating nanovoids within the nanosheets. Simultaneously, the dissolved Mo species redeposit *in situ* as ultrafine amorphous MoO_3_ nanoparticles embedded within the crystalline NiP matrix, generating a dense array of crystalline-amorphous nanointerfaces throughout the material. This stands in contrast to the sparse interfaces produced by conventional surface-confined reconstruction. Spatial confinement offers a complementary route to guide reconstruction. Liang *et al.* designed a ZnO@CoMoO_4_ core–shell heterostructure for alkaline HER.^108^ Under cathodic polarization, the CoMoO_4_ shell reconstructs into Co(OH)_2_, while the ZnO core dissolves to generate Zn species in the alkaline electrolyte. The core–shell architecture serves a critical spatial function to confine the dissolved Zn species within the interfacial region. This confinement function creates a local concentration gradient that drives their short-range migration into the evolving Co(OH)_2_ phase, resulting in Zn-doped Co(OH)_2_ as the reconstructed catalytic phase. Therefore, the pre-designed core–shell interface not only spatially confines the dissolved species but, more critically, guides the fundamental chemical composition and electronic structure of the final active species through its unique coupled dissolution-reconstruction kinetics.^109^

Beyond composition and crystal structure, the reconstruction pathway can also be triggered and guided through deliberate electrochemical activation protocols.^110,111^ Zhao *et al.* developed a Ru/WO_*x*_ catalyst that undergoes *in situ* oxidation of Ru during HER operation, forming a RuO_*x*_/Wo_2_ interface (Fig. 6g) that optimizes the electronic structure and strengthens hydrogen spillover from Ru sites to the WO_*x*_ support (Fig. 6h).^112^ Chen *et al.* reported a Cr-doped Ni_3_S_2_ catalyst that undergoes surface reconstruction during *in situ* cathodic activation, introducing sulfur vacancies and oxygen species onto the surface to strengthen OH^−^ desorption and optimize hydrogen intermediate desorption kinetics.^113^ In both cases, the activation process serves as a programmable trigger for reconstruction. The applied potential program dictates the extent and nature of structural evolution, converting a moderately active pre-catalyst into a highly active reconstructed phase through the targeted introduction of vacancies, oxygen species, or new interfacial phases.

Across these diverse strategies, the reconstruction pathway of a pre-catalyst is not determined solely by the electrochemical conditions. It is fundamentally encoded in its initial structure. This encoding can take different forms, including compositional heterogeneity, electronic structure tuning, crystallographic feature control, or the application of an activation protocol. In each case, the initial structure pre-selects the thermodynamic driving forces and kinetic pathways that the material will follow under electrochemical bias. The active phases ultimately generated from these diverse pathways can be categorized into several typical forms, including oxyhydroxides, enriched alloy surfaces, single-atom or cluster-support composite structures, and heterointerfaces. Table 1 summarizes representative pre-catalyst systems based on category, illustrating how the initial material characteristics preset the reconstruction pathway and ultimately determine the final active phase. Note that the listed HER performances are provided to illustrate the relationship between reconstruction pathways and catalytic behaviors rather than to establish a direct performance ranking. Therefore, HER reconstruction should be understood as a process governed by thermodynamic driving forces together with kinetic constraints. This insight shifts the design paradigm from stabilizing a catalyst against change to encoding a desired evolutionary trajectory into its initial architecture.

**Table 1:** Comparison of reconstruction pathways, active phases, and HER performance of representative HER pre-catalysts

Category	Catalyst	Catalyst loading (mg cm^−2^)	Active phase	HER activity	Ref.
Sulfide	SV-Co_9_S_8_	2	Co(OH)_2_/SV-Co_9_S_8_ heterostructure	*η*_10_ = 217 mV (1 M KOH)	72
	S-Co_3_O_4_/CC	—	Co-Co^*δ*+^S_*x*_	*η*_10_ = 119 mV (1 M KOH)	115
	M-MoS_2_	0.56	V_MoS5_-MoS_2_	*η*_10_ = 210 mV (0.5 M H_2_SO_4_)	116
	Ni_0.8_Fe_0.2_S_2_	—	Ni_3_S_2_	*η*_10_ = 121 mV (1 M KOH)	117
Phosphides	NCMP(MXene)	22	NiCoMn(OH)_2_	*η*_10_ = 100 mV (1 M KOH)	62
	Se-CoP/CC	1.14	Co(OH)_2_/CoNC-P-Se	*η*_100_ = 79 ± 2 mV; *η*_500_ = 115 ± 4 mV (1 M KOH)	118
	Co@CoFe–P NBs	0.3	P–Co–O–Fe–P (M^0^/M^+^)	*η*_10_ = 83 mV (0.5 M H_2_SO_4_)	119
				*η*_10_ = 104 mV (1 M KOH)	
				*η*_10_ = 150 mV (1 M PBS)	
	MoO_3_/Mo-NiP (NF)	—	Amorphous MoO_3_/crystalline NiP heterointerface	*η*_10_ = 26 mV; *η*_500_ = 290 mV (1 M KOH)	120
	CNFMPO	—	M(OH)_2_/M-OOH	*η*_10_ = 43 mV (1 M KOH); *η*_10_ = 73 mV (1 M KOH + 0.5 M NaCl)	121
	Ni-CoP/Co_2_P@NC	0.283	Ni-CoP/Co_2_P@NC	*η*_10_ = 117 mV (HER, 1 M KOH)	122
Single atoms/clusters	Co(OH)_2_@	0.63	Co(OH)_2_@ Co_2_Mo_3_O_8_ heterostructure	*η*_10_ = 85 mV; *η*_50_ = 161 mV (1 M KOH)	123
	Co_2_Mo_3_O_8_				
	Mo-RuCoO_*x*_	1.1	Mo-RuCoO_*x*_|Co(OH)_2_ heterostructure	*η*_10_ = 41 mV; *η*_100_ = 77 mV (1 M KOH)	124
	NM_8_-R	—	MoNi_4_	*η*_10_ = 23 mV (0.5 M H_2_SO_4_)	125
	LaRuSi_3_@Ru	4.2	Ru clusters/LaRuSi_3_	*η*_10_ = 45 mV (1 M KOH)	126
	Ru-LC-Ni(OH)_2_	1.21	Ru-Ni(OH)_2_	*η*_10_ = 9 mV (1 M KOH)	127
Heterojunctions/core–shell	Ni_3_Se_2_/NiO	—	Ni_3_Se_2_/NiO heterointerface	*η*_10_ = 70 mV (1 M KOH)	105
	Ru_2_Pd_1_O_*x*_-350 HNFs	∼1	RuO_2_/Pd heterointerface	*η*_10_ = 11 mV; *η*_1000_ = 120 mV (1 M KOH)	128
	Cu/Fe_3_O_4_	2.53	Cu-FeOOH/Fe_3_O_4_ heterointerface	*η*_100_ = 129 mV; *η*_500_ = 285 mV (1 M KOH)	129
	Reconstructed Zn-Co(OH)_2_	—	Zn-doped Co(OH)_2_	*η*_10_ = 40 mV (1 M KOH)	130
	Ni/WO_*x*_-MoM	1.8	Ni/WO_*x*_-MoO_4_^2−^	*η*_1000_ = 183 mV (1 M KOH)	131
	Cu_3_P NWs@CF	0.266	PtCu/Cu_3_P	*η*_10_ = 37.8 mV (0.5 M H_2_SO_4_)	132
	MoNi_4_@NF	1.4 ± 0.06	Mo-NiOOH	*η*_200_ = 80 mV (1 M KOH)	133
	HEAs-Co_2_RuO_4_	—	HEAs-Co_2_RuO_4_	*η*_10_ = 11.8 mV (1 M KOH)	134
	6H-BaRuO_3_	1	Ru_*x*_O_*y*_/6H-BaRuO_3_	*η*_10_ = 11 mV (1 M KOH)	114
	AC-STRO	0.382	Ru/SrTi_0.5_Ru_0.5_O_3_	*η*_10_ = 18 mV (1 M KOH)	135
	Zn-Ni_2_P/FeOOH	—	Ni_2_P/FeOOH	*η*_10_ = 11 mV (1 M KOH)	136
	F_d_-PTS-NLM	—	NiFe LDH/MOF-74	*η*_10_ = 150 mV (1 M KOH)	137

## 3 Driving forces of reconstruction and rational design strategies

Understanding the driving forces that govern reconstruction is essential for translating mechanistic insight into design principles. Phenomenological observation alone is insufficient to underpin a theoretical framework for rational catalyst design. The key question concerns what forces dictate that reconstruction proceeds along a particular pathway and how these insights can guide the steering of reconstruction toward a desired direction. Reconstruction kinetics and HER kinetics operate on different timescales and are governed by distinct physical processes. Reconstruction kinetics involve structural evolution of a catalyst, including the reconstruction potential, environmental pH, reconstruction time, *etc.* HER kinetics, by contrast, concern the surface-mediated catalytic cycle, including charge transfer at the electrode–electrolyte interface, intermediate adsorption and desorption, and product evolution. Therefore, the key to understanding the reconfiguration process of the HER pre-catalyst lies in analyzing which factors influence this reconfiguration process. This chapter addresses these issues in two steps. It first dissects the extrinsic electrochemical conditions and intrinsic material properties that collectively govern HER reconstruction. On this basis, it then elaborates the rational design strategies that have emerged from this mechanistic understanding.

### 3.1 Driving forces of reconstruction

The reconstruction of a pre-catalyst is not a random structural collapse, but rather the outcome of the synergistic interplay between the material's intrinsic evolutionary propensity and the selection pressure imposed by the electrochemical environment. Intrinsic and extrinsic factors are not independent variables; they exist in a state of dynamic mutual feedback. The applied potential and pH environment provide the electrochemical driving force to overcome kinetic barriers, while prolonged polarization allows progressive mass transport and surface restructuring. Also, instinct bond covalency and defect density determine the material's susceptibility to external perturbations. Together, these factors influence the starting point and pathway of reconstruction, while the final reconstructed state is also constrained by kinetic accessibility and the local electrochemical environment. It should be noted that the observed final reconstructed structure may be kinetically trapped or dynamically maintained, rather than representing a global equilibrium state. This section generalizes the driving forces into three categories, each operating at a distinct level: the external electrochemical environment, the internal electronic structure, and the atomic-scale structural imperfections.

#### 3.1.1 External electrochemical environment: pH, potential, and time

The external electrochemical environment imposes thermodynamic and kinetic constraints that define the feasible reconstruction pathways. These constraints determine not only whether reconstruction occurs, but also which pathway the system follows and where it terminates. The electrolyte pH, applied potential, and polarization time each govern a distinct aspect of this process, and their interplay collectively dictates the final structure of the reconstructed catalyst.^138–142^

**Fig. 7:**
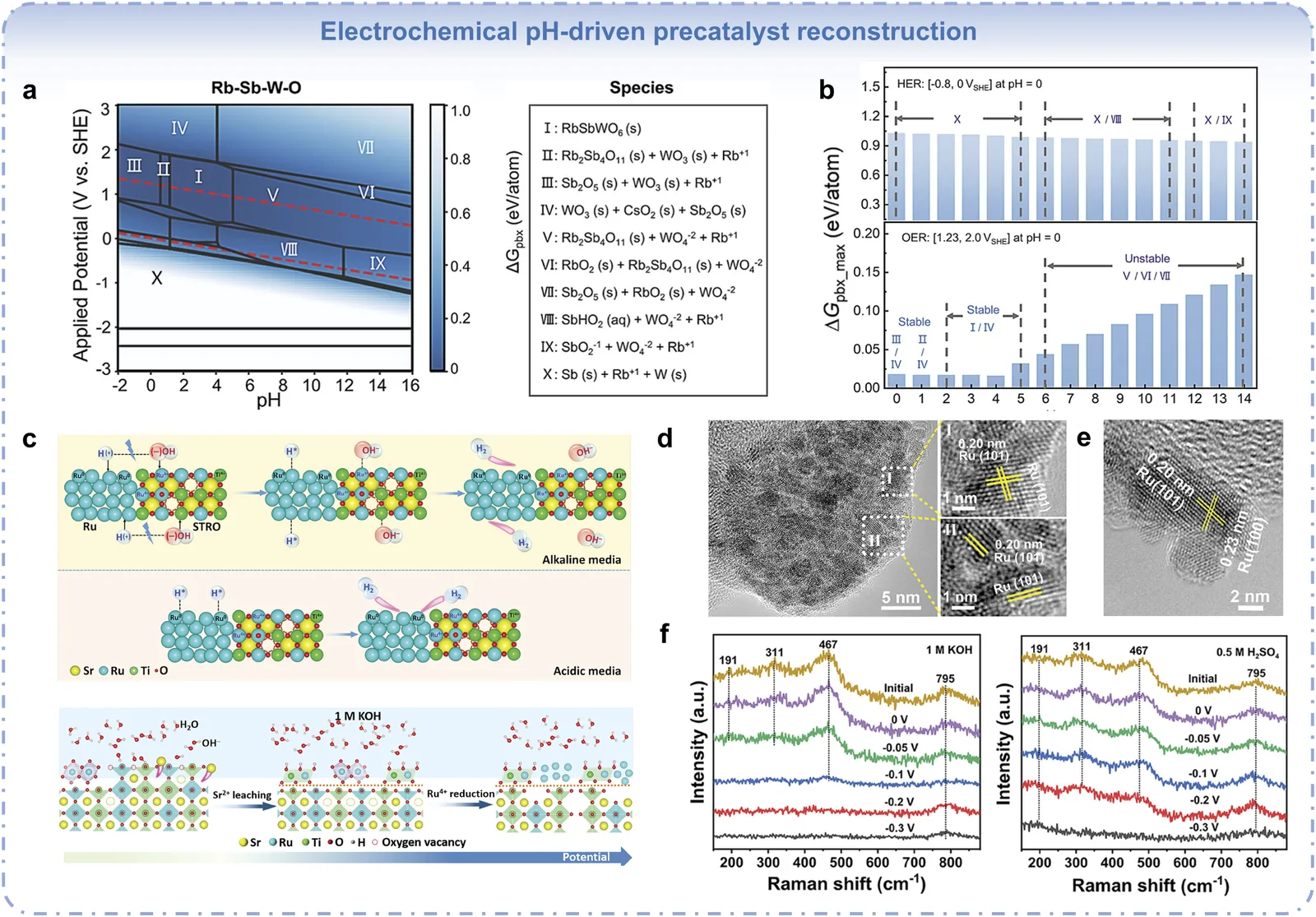
(a) Bulk Pourbaix diagram of the Rb–Sb–W–O system under OER conditions. (b) Bulk Pourbaix diagram of the Rb–Sb–W–O system under HER conditions.^144^ Copyright 2025, American Chemical Society. (c) Schematic illustration of the electrochemical reconstruction on the surface of STRO in alkaline media. (d) HRTEM image of AC-STRO after 10 k CV cycles in 1 M KOH. (e) HRTEM image of AC-STRO after 10 k CV cycles in 0.5 M H_2_SO_4_. (f) *In situ* Raman spectra of STRO during the HER in 1 M KOH and 0.5 M H_2_SO_4_.^145^ Copyright 2024, Wiley-VCH.

The electrolyte pH exerts a pronounced influence on the surface reconstruction pathway by altering both the proton source and the concentration of hydroxide ions. In alkaline media, OH^−^ can act as a nucleophile attacking metal center, inducing surface oxidation or hydroxylation. Under acidic conditions, the high proton concentration and strong corrosiveness drive metal dissolution or hydride formation. Liu *et al.* investigated the reconstruction difference of RuNi alloys in acidic and alkaline media. In alkaline electrolyte, surface Ni atoms undergo hydroxylation to form Ru-Ni(OH)_2_ bifunctional active sites; in acidic electrolyte, Ni is selectively etched away, leaving monometallic Ru active sites.^143^ This is not a material-specific phenomenon but a general thermodynamic consequence of the Pourbaix diagram of each element. The stable phase of a metal at a given pH and potential is dictated by its equilibrium with either OH^−^ or H^+^. The same material can therefore undergo fundamentally different reconstruction pathways simply by changing the electrolyte pH, yielding distinct active phases that are optimized for different operating conditions. A compelling demonstration comes from a study on RbSbWO_6_ as an acidic water splitting catalyst.^144^ Bulk Pourbaix analysis reveals that RbSbWO_6_ remains thermodynamically stable as a solid phase only within a narrow pH window of 2 to 5 at oxidation potentials (Fig. 7a). At pH 0–1, it decomposes into WO_3_, CsO_2_, and Sb_2_O_5_; above pH 6, no solid phase is stable. At HER potentials, no solid phase is thermodynamically stable across all pH values (Fig. 7b). This analysis establishes that pH fundamentally presets the thermodynamic feasibility of reconstruction. The same material can be stable under one condition but undergo complete decomposition under another, and this behavior can be systematically predicted through Pourbaix analysis rather than observed as an unpredictable corrosion process. Notedly, conventional Pourbaix diagrams mainly describe bulk equilibrium phases and therefore provide thermodynamic guidance rather than a direct prediction of the reconstructed active states in nanoscale HER catalysts. The experimentally observed structures may deviate from the equilibrium phases predicted by Pourbaix analysis because surface energies, adsorbate stabilization, local interfacial environments, kinetic barriers, and metastable configurations can significantly influence reconstruction under electrochemical conditions. The pH-dependent divergence is also proved by the contrasting reconstruction behavior of Ti-doped strontium ruthenate perovskite (SrTi_0.5_Ru_0.5_O_3_, STRO) in electrolytes of different pH (Fig. 7c).^145^ In an alkaline medium, STRO undergoes rapid and deep surface reconstruction, involving Sr^2+^ leaching, *in situ* reduction of Ru^4+^ to metallic Ru nanoparticles, and the formation of amorphous RuTi oxyhydroxide layer (Fig. 7d and e). This stands in stark contrast to the sluggish evolution observed in the acidic medium. *In situ* Raman spectroscopy captured this kinetic divergence, with the characteristic Ru–O vibration decaying rapidly at alkaline potential while being significantly retarded in acid (Fig. 7f). Fan *et al.* systematically investigated the reconstruction process of porous CoW sulfides for pH-universal HER.^146^ Using *operando* X-ray diffraction and X-ray absorption spectroscopy, they found that the structural transformation of CoW sulfides is strongly dependent on the electrolyte pH. Under acidic conditions, the reformation merely involves the surface Co species being transformed into oxides or hydroxides, while under neutral and alkaline conditions, the interstitial cobalt species will be reconstructed to form CoO. These examples establish that pH is not merely a background parameter but an active determinant of the reconstruction rate, depth and pathway.

**Fig. 8:**
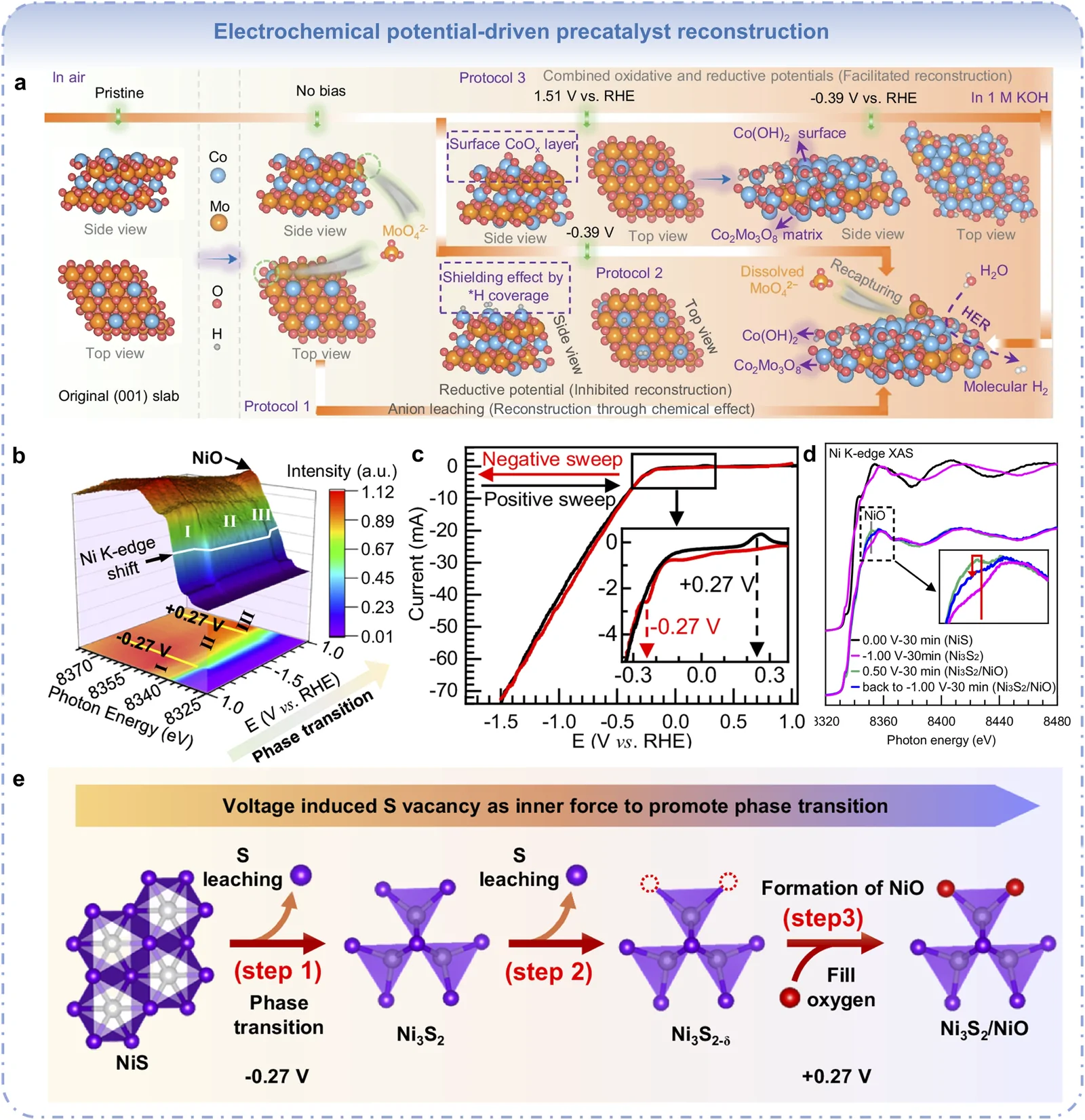
(a) Schematic illustration of the programmed protocols for developing Co(OH)_2_@Co_2_Mo_3_O_8_ structures and elucidating the potential-dependent structure evolution mechanism of the Co_2_Mo_3_O_8_ precatalyst.^123^ Copyright 2025, Springer Nature. (b) *Operando* Ni K-edge XAS spectra of NiS as a function of applied voltages. (c) Corresponding CV profile used for the *operando* XAS measurement. (d) *Operando* Ni K-edge XAS spectra of NiS during chronoamperometry at four consecutively applied potentials. (e) Schematic illustration of the phase transition mechanism of NiS during the HER.^150^ Copyright 2026, Wiley-VCH.

Except for electrolyte pH, the applied potential also determines the thermodynamic driving force and the final product of reconstruction. Each reconstruction step, whether dissolution, phase transformation, or redeposition, is associated with a characteristic potential window.^147–149^ Within a given potential window, the pre-catalyst evolves toward the thermodynamically most stable phase under these conditions. As the potential crosses different threshold values, the dominant reconstruction pathway shifts accordingly. This potential-dependent behavior is systematically illustrated by Zhu *et al.*^123^ on Co_2_Mo_3_O_8_ as a model pre-catalyst. As shown in Fig. 8a, under cathodic polarization, Co_2_Mo_3_O_8_ undergoes reconstruction to generate an electrochemically stable Co(OH)_2_@Co_2_Mo_3_O_8_ heterostructure, while simultaneously releasing Mo species into the electrolyte in the form of MoO_4_^2−^. Importantly, the applied negative potential further induces the conversion of MoO_4_^2−^ in the electrolyte to Mo_2_O_7_^2−^, which enhances proton adsorption and H_2_ desorption kinetics on the catalyst surface. The difference in reconstruction products at different potentials arises from the fact that the thermodynamic stability of each possible product varies with potential. At a given potential, the system selectively evolves toward the favorable thermodynamic phase. Among all candidates, and as the potential shifts, the relative stability order of these candidates changes, yielding distinctly different final structures. Ding *et al.* further elucidated the potential-driven phase transformation kinetics of NiS, which undergoes a phase transition from NiS to Ni_3_S_2_ at reductive potentials and forms a mixed phase upon back-scanning (Fig. 8b and c).^150^ The real-time evolution of XAS spectra also demonstrated that the reconstructed Ni_3_S_2_/NiO mixed phase could stably exist under long-term hydrogen evolution reaction conditions (Fig. 8d). The reconstruction was deconstructed into a three-step potential-programmed mechanism (Fig. 8e): NiS is first reduced and transformed into Ni_3_S_2_, followed by S leaching to form vacancies (Ni_3_S_2−*δ*_) at more negative potentials, and finally these vacancies are filled by O oxidation, creating highly active Ni_3_S_2_/NiO heterointerfaces.

Also, applied potential can modulate the electronic state of surface species.^151^ Fan *et al.*, using *in situ* Raman spectroscopy, observed that during the HER on S–Co_3_O_4_, the Co–S stretching vibration peak undergoes a continuous blue shift as the potential moves cathodically, indicating dynamic evolution of the electronic state of surface-adsorbed sulfur species.^115,151^ Combined with *in situ* XANES, they confirmed the ultimate formation of a metallic Co framework composited with low-nuclearity CoS_*x*_ adsorbed species. This work demonstrates that potential can continuously drive the material toward an optimal catalytic configuration through modulation of surface chemical states. Collectively, this potential-dependent behavior explains why the same pre-catalyst can yield different active phases under different polarization conditions and why certain reconstructed structures can be selectively obtained by choosing the appropriate potential range.

**Fig. 9:**
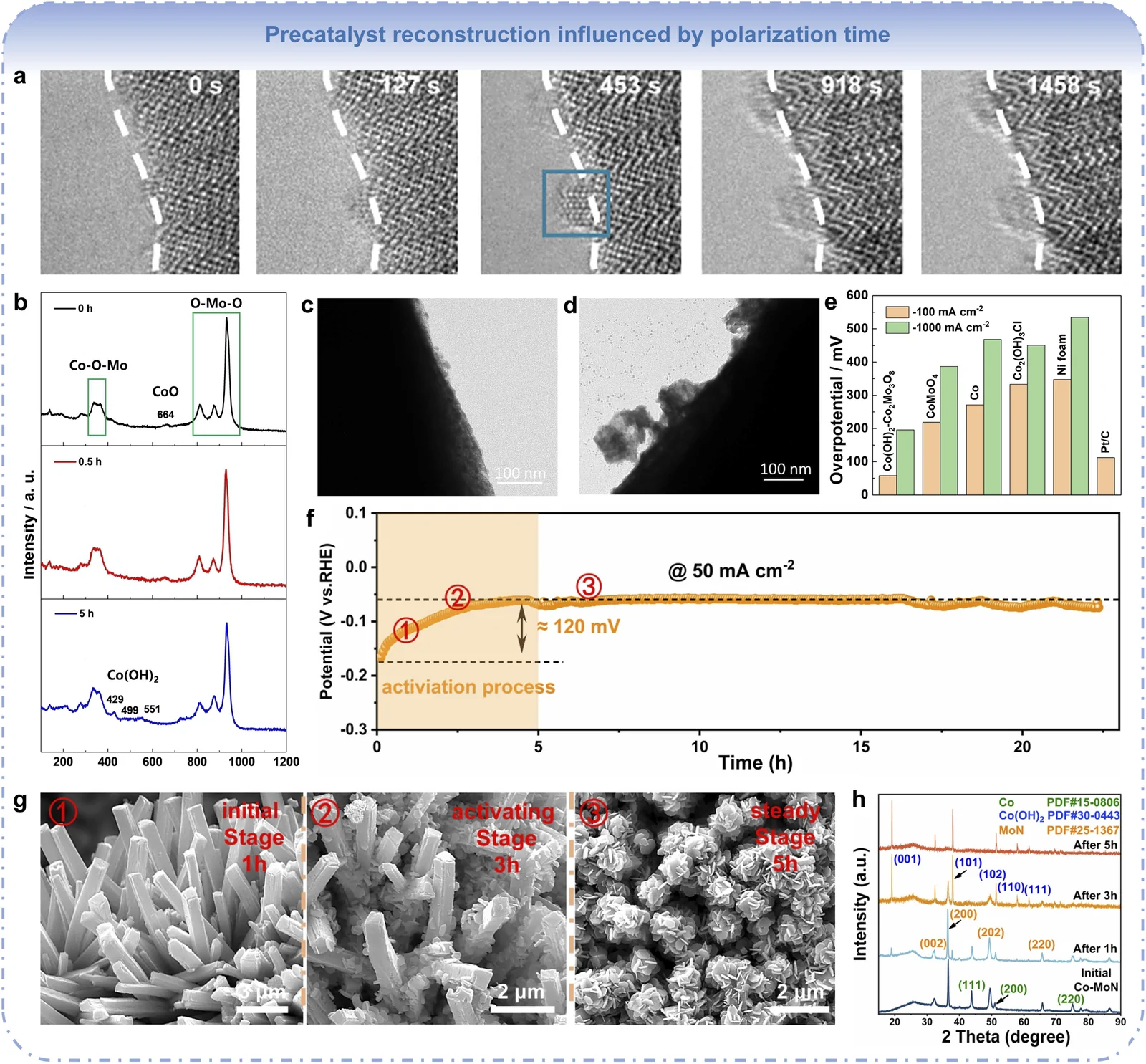
(a) HRTEM images of initial BRO showing *in situ* Ru exsolution over time under TEM observation.^106^ Copyright 2026, American Chemical Society. (b) Raman spectra of CoO-Co_2_Mo_3_O_8_ after different durations of surface reconstruction. (c and d) SEM images of CoO-Co_2_Mo_3_O_8_ and Co(OH)_2_-Co_2_Mo_3_O_8_. (e) Overpotentials of Co(OH)_2_-Co_2_Mo_3_O_8_.^152^ Copyright 2025, Wiley-VCH. (f) Chronopotentiometry curve of the Co-MoN electrode during electrochemical activation. (g) SEM images and (h) XRD patterns of Co-MoN at different reconstruction stages.^153^ Copyright 2025, Elsevier.

Whereas pH and potential define the thermodynamic tendency to overcome kinetic barriers, polarization time determines how far the kinetically accessible structural evolution can proceed. Short-term polarization may preserve metastable intermediates, while prolonged operation allows continued mass transport and surface restructuring to form a thermodynamically favorable or kinetically trapped phase. This time-dependent progression is exemplified in the work of Zhao *et al.* on ruthenate perovskites (ARuO_3_, where A = La, Ca, Sr, Ba). Among the series, BaRuO_3_ (BRO), with its O 2p band center closest to the Fermi level, exhibits the most pronounced surface reconstruction, forming a Ru-exsolved Ru/BRO heterostructure through potential-induced activation.^106^ High-resolution transmission electron microscopy captured the dynamic Ru exsolution process in real time (Fig. 9a). The reconstruction starts from an initially smooth surface, and Ru nanoclusters progressively nucleate and grow with increasing polarization time, accompanied by the generation of oxygen vacancies in the oxide framework, with the extent of Ru exsolution increasing almost synchronously with time. The reconstructed heterostructure achieves an approximately 120-fold enhancement in HER activity compared to the pristine material, with a mass activity 1.68 times that of commercial Pt/C. The time-dependent progression of reconstruction is further illustrated in the work of Lu *et al.* on a CoO-Co_2_Mo_3_O_8_ integrated electrode for alkaline HER.^152^ The CoO component undergoes surface reconstruction over time, gradually transforming into Co(OH)_2_ during HER operation, while the Co_2_Mo_3_O_8_ framework remains structurally intact (Fig. 9b). TEM images reveal the morphological transformation from CoO nanoparticles loaded on Co_2_Mo_3_O_8_ nanorods to uniformly coating Co(OH)_2_ nanosheets after sufficient polarization (Fig. 9c and d). The reconstructed Co(OH)_2_-Co_2_Mo_3_O_8_ interface achieves overpotentials of only 57.8 mV and 195.8 mV at 100 mA cm^−2^ and 1000 mA cm^−2^, respectively, with stable operation for 200 hours at high current densities (Fig. 9e).

Another important factor is the sequence relationship with the HER and reconstruction. Reconstruction may occur before measurable HER activity as an activation process, concurrently with the HER as the surface evolves dynamically, or continuously during steady-state operation as the structure slowly drifts under sustained polarization. Pi *et al.* further probed the time-driven reconstruction from a dissolution-redeposition perspective using Co/MoN as a model pre-catalyst.^153^ With increasing polarization time, the Co/MoN catalyst shows significantly reduced overpotential of the HER by about 120 mV and gradually stabilized (Fig. 9f). This indicates that the catalyst undergoes a continuous reconstruction process over time and eventually forms an active phase. SEM images capture the morphological evolution from tetragonal Co/MoN nanoarrays at 1 h to Co(OH)_2_ nanosheets at 5 h (Fig. 9g). Quasi-*in situ* XRD reveals that Co first dissolves as Co^2+^ and then redeposits directionally as Co(OH)_2_ nanosheets, guided by lattice matching with the MoN (002) facet (Fig. 9h). Prolonged polarization thus enables the system to overcome kinetic barriers, driving reconstruction toward a kinetically complete steady state. It is noteworthy that the key consideration is whether the reconstructed composition remains stable at the applied HER potential and whether the reconstruction kinetics reach a dynamic equilibrium under sustained polarization, such that the catalyst achieves a steady-state structure with stable performance. These examples collectively demonstrate that reconstruction is not an instantaneous event but a kinetically controlled process, where the extent of structural evolution and the resulting catalytic performance depend critically on the duration of polarization.

#### 3.1.2 Intrinsic material properties: electronic structure and lattice defects

In an identical electrochemical environment, different materials often follow dramatically different reconstruction pathways. This divergence originates from intrinsic properties such as the d-band center position, metal-anion bond covalency, and lattice defects.^154–157^ These properties dictate both the thermodynamic driving force for reconstruction and the kinetic barriers that must be overcome. They also determine how sensitively the material responds to external perturbations such as pH and potential.

One of the important intrinsic factors is the electronic structure, particularly the band center position and bond covalency. The band center dictates the adsorption strength of intermediates and the stability of metal-anion bonds during reconstruction.^158,159^ Alloying and doping modulate the d-band center by altering electronic occupation and orbital hybridization, thereby presetting the reconstruction propensity. This electronic structure-reconstruction relationship is illustrated in the work of Zhang *et al.* on Se-doped CoP.^118^ The introduction of lattice Se modulates the bond covalency of Co–P, accelerating the reconstruction of Se–CoP during the HER and yielding a heterostructure with crystalline Co(OH)_2_ and stable Co nanoclusters. This case demonstrates that anion doping presets both the reconstruction rate and the crystallinity of the final product. Li *et al.* revealed that Ce-4f states modulate Co sites through f-p-d gradient orbital coupling.^160^ Under HER conditions, the Ce-4f state induces parallel spin alignment of electrons on surrounding Co sites, optimizing H* adsorption; under OER conditions, the same Ce-4f band acts as a sacrificial band that protects the Co–O bond covalency in the oxidation potential range. This potential-dependent role reversal exemplifies how the d-band center is not a static property but a dynamic entity that responds to external potential.

**Fig. 10:**
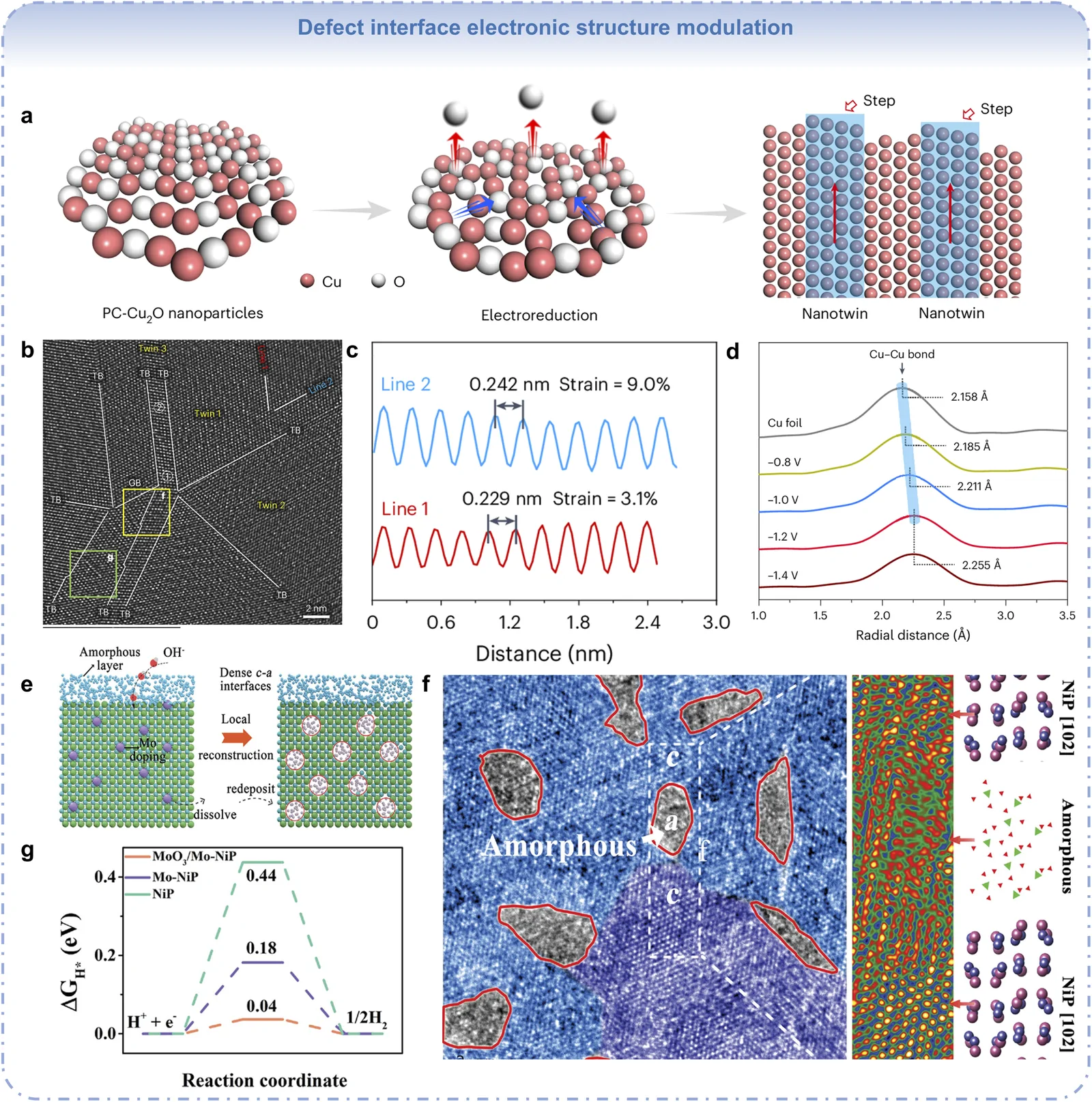
(a) Schematic illustration of the synthesis and crystal structure of the DNTs-Cu catalyst. (b) HRTEM image of a PC-Cu_2_O nanoparticle showing polycrystalline nanograins. (c) Lattice distance scans along lines 1 and 2, showing the calculated tensile strain compared with that of the standard Cu lattice. (d) Radial distribution functions of DNTs-Cu catalysts obtained at different electroreduction potentials and Cu foil.^164^ Copyright 2025, Springer Nature. (e) Schematic diagram of dynamic construction of dense crystalline-amorphous nano-interfaces during local reconstruction. (f) HRTEM image of MoO_3_/Mo-NiP/NF showing dense crystalline-amorphous interfaces. (g) Free energy diagrams of adsorbed H* intermediates.^107^ Copyright 2024, Wiley-VCH.

While the electronic structure determines the thermodynamic propensity for reconstruction, lattice defects define the spatial and kinetic pathways through which reconstruction proceeds. Defects such as grain boundaries, dislocations, and vacancies are high-energy sites that lower the kinetic barrier for atomic migration, phase transformation, and elemental leaching.^161–163^ Li *et al.* revealed a grain-boundary-driven electro-reductive reconstruction mechanism using polycrystalline Cu_2_O.^164^ As shown in Fig. 10a, during electro-reduction to pure Cu, the high density of grain boundaries in the precursor served as preferential sites for phase transformation due to a significantly lower Cu diffusion barrier compared to the bulk. The subsequent grain merger locked in distorted nanotwins and edge dislocations with local tensile strain (Fig. 10b), with local tensile strain up to 9.0% (Fig. 10c). EXAFS confirms the resulting increased Cu–Cu bond length and reduced coordination number, synergistically modulating the d-band center (Fig. 10d). Control experiments confirm the causal role of these defects, where pre-annealing the Cu_2_O to eliminate grain boundaries abolished these defect configurations and drastically reduced HER activity. While the Cu_2_O study leverages native grain boundaries, deliberate engineering through doping offers another level of control. Nie *et al.* demonstrated this concept using Mo-doped NiP as a pre-catalyst, revealing how doping-induced local lattice mismatch directs the reconstruction pathway.^107^ Under polarization, Mo preferentially leaches from the bulk, creating voids that direct the *in situ* formation of amorphous MoO_3_ nanoparticles and constructing a dense crystalline-amorphous nanointerface (Fig. 10e and f). DFT calculations (Fig. 10g) reveal that this defect interface drives electron redistribution through a Mo–O–P bridging mechanism, optimizing Δ*G*_H^*^_ to near zero (0.04 eV), whereas Mo-doping alone only achieved 0.18 eV. The electronic modulation at the defect interface is therefore the decisive factor behind the leap in HER performance.

The reconstruction of a pre-catalyst is governed by the interplay between extrinsic driving forces and intrinsic material properties. The reconstruction is the result of extrinsic driving forces (potential, pH, and polarization time) exerting selection pressure on the intrinsic evolutionary propensity (electronic structure and defects), with the two engaged in dynamic mutual feedback.^165,166^ External conditions such as pH, potential, and time define the thermodynamic feasibility and kinetic accessibility of reconstruction pathways. Intrinsic properties such as the d-band center and lattice defects determine which pathway the material follows and how far the reconstruction proceeds. The key insight is that extrinsic conditions do not simply trigger reconstruction but continuously modulate the electronic manifestation of intrinsic properties; likewise, intrinsic properties are not a static blueprint but respond dynamically to external perturbations.

### 3.2 Rational design strategies

The ultimate purpose of elucidating the driving forces and evolutionary pathways of reconstruction is to translate these insights into design principles for pre-catalyst engineering. The rational design strategies developed to date share a common core. The formation pathway of the target active phase is pre-encoded into the initial catalyst structure in the form of an atomistic program. It should be noted that most reported reconstruction-guided designs remain at the stage of retrospective rationalization, where the initial structural or compositional features are analyzed after reconstruction to identify factors associated with the observed evolution. In contrast, prospective pre-encoding requires the evaluation of quantitative descriptors before synthesis to anticipate possible reconstruction pathways. Therefore, although reconstruction-guided design provides a conceptual route toward selective evolution of active states, fully predictive identification of precursor compositions and architectures remains an open challenge.

#### 3.2.1 Defect and vacancy engineering

As a means of precise tailoring at the atomic scale, defect and vacancy engineering operates through pre-constructing vacancies or introducing heteroatoms into the parent material. This approach can lower the kinetic barriers to reconstruction and reshape the electronic configuration of the material, thereby inducing the formation of thermodynamically favorable and highly active phases.^124,167,168^

**Fig. 11:**
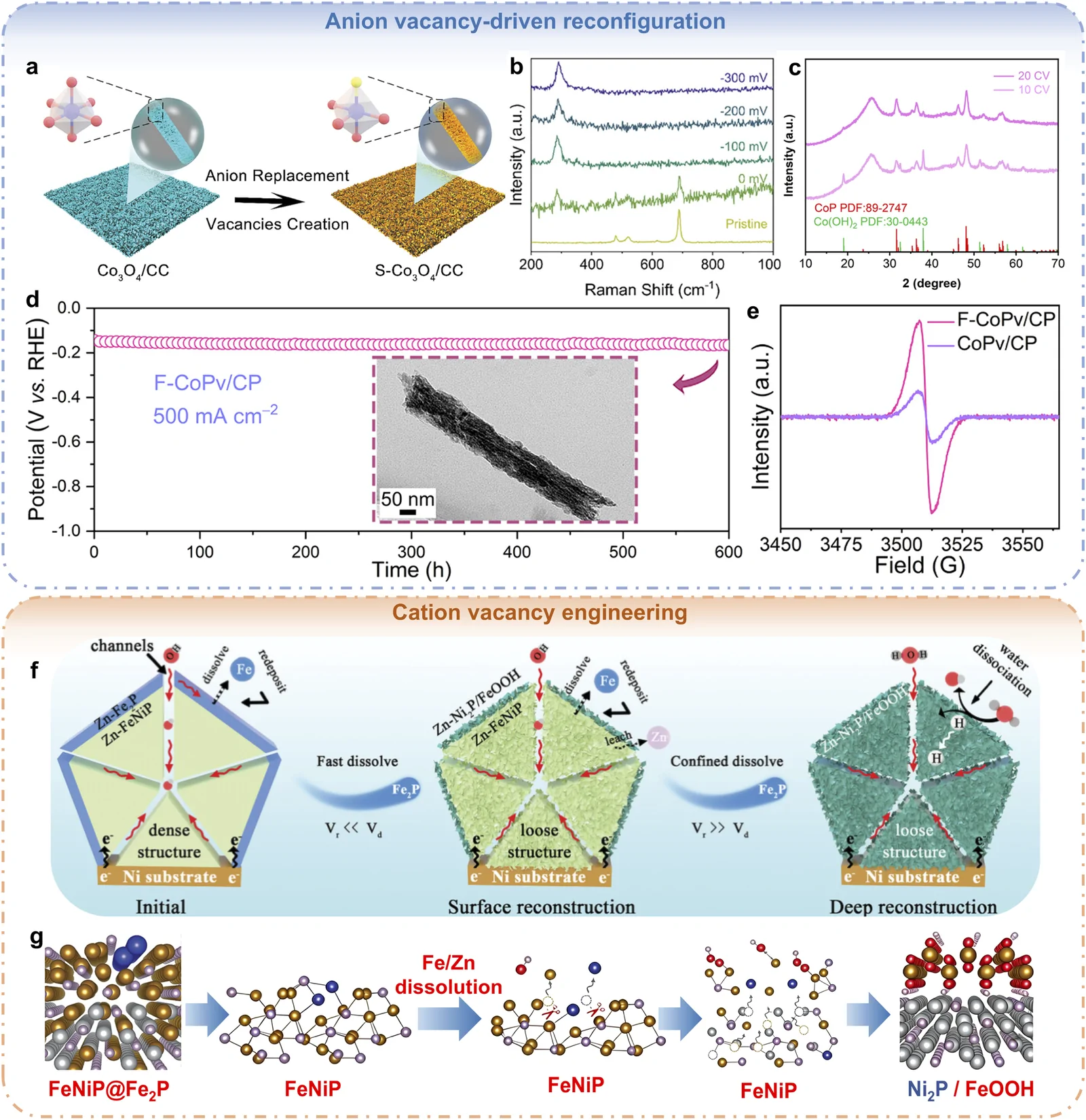
(a) Schematic illustration of the topotactic transformation from Co_3_O_4_ to S-Co_3_O_4_*via* H_2_S treatment. (b) *In situ* Raman spectra of S-Co_3_O_4_/CC recorded during the HER.^115^ Copyright 2024, Wiley-VCH. (c) *Ex situ* XRD patterns of Br–CoP during HER reconstruction.^169^ Copyright 2025, Wiley-VCH. (d) Chronopotentiometry curve of F-CoP/CP at 500 mA cm^−2^ for over 600 h. (e) EPR spectra of F-CoP/CP and CoP/CP.^170^ Copyright 2025, American Chemical Society. (f) Schematic illustration for the cathodic reduction reconstruction of Zn-FeNiP@Zn-Fe_2_P during the HER. (g) The microstructure reconstruction mechanism of the “dissolution-redeposition” process determined by theoretical calculations.^171^ Copyright 2024, Wiley-VCH.

Anion vacancy strategies operate on a core logic of weakening metal-anion bond strength to lower the reconstruction energy barrier. Fan *et al.* demonstrated this concept using S-doped Co_3_O_4_, where lattice O^2−^ was partially replaced by S^2−^ through H_2_S treatment, synchronously generating abundant oxygen vacancies (Fig. 11a).^115^ The introduced oxygen vacancies and S coordination atoms can rapidly induce the *in situ* electrochemical reconfiguration of S–Co_3_O_4_. Under HER conditions, *operando* Raman spectroscopy (Fig. 11b) confirmed that the Co ions in S–Co_3_O_4_ are reduced to metallic Co, while S is dispersed on the surface of the metallic Co in the form of S^2−^, forming reconstructed low-nuclearity CoS_*x*_ clusters on the metallic Co surface system. Extending this logic to transition metal phosphides, P vacancies play a dual role as lattice defects exposing undercoordinated metal sites and as agents that accelerate reconstruction by weakening lattice stability. Yao *et al.* dissected this mechanism using Br-doped CoP.^169^ The introduction of Br has altered the electronic structure of the Co site and induced the formation of the P-poor phase of CoP. This anion vacancy engineering is more likely to lead to reconstruction during the HER process. As evidenced by the *ex situ* XRD pattern in Fig. 11c, under cathodic activation and HER conditions, Br–CoP undergoes irreversible surface reconstruction to form a CoP–Co(OH)_2_ reconstructed interface, which serves as the active site during the HER. In stark contrast to this accelerated reconstruction, Li *et al.* demonstrated that defect engineering can also result in extraordinary structural stability.^170^ F doping lowered the P vacancy formation energy by 0.87 eV, allowing abundant P vacancies to be generated under mild conditions. After a 600 h chronopotentiometry test, the morphology remained largely intact and the P vacancy signal persisted (Fig. 11d and e), revealing that the active sites originated from the intrinsic structure of the pre-catalyst rather than from deep reconstruction.

Compared with anion vacancies, the formation energy of cation vacancies is usually higher and is difficult to be controllably introduced under normal conditions.^172–174^ However, it is precisely this high formation energy that endows cation vacancies with unique scientific value. Once they form, the disturbance they cause to the electronic structure of the material is often profound and persistent. Nie *et al.* demonstrated this using Zn^2+^ doping in FeNiP@Fe_2_P.^171^ DFT calculations (Fig. 11f) revealed that Zn introduction stretched both Fe–P and Zn–P bonds, drastically reducing structural stability. Under cathodic polarization, the weakened bonds preferentially broke, releasing Fe^2+^/Zn^2+^/P^4−^ ions, with Fe^2+^ redepositing as amorphous FeOOH through a dissolution-redeposition mechanism (Fig. 11g). This work shows that Zn^2+^ induces bulk-level deep reconstruction, breaking through the limitation of conventional surface-confined reconstruction. In a complementary approach, Zhang *et al.* demonstrate that the incorporation of Mo into Ru–Co_3_O_4_ enables a reconstruction pathway in which the controllable dissolution of Mo couples with the generation of oxygen vacancies.^124^ During HER operation, the leached Mo atoms create vacancies that facilitate surface reconstruction into Co(OH)_2_, while the accompanying oxygen vacancies synergistically optimize the adsorption energy of hydrogen intermediates. This combined electronic and vacancy engineering enables the Mo-RuCoO_*x*_ nanoarrays to achieve remarkably low overpotentials of 41 mV at 10 mA cm^−2^ for the HER. In summary, the common essence of cation vacancy strategies lies in creating metal-deficient sites within the lattice to weaken bonding strength, thereby providing kinetic channels for electrolyte penetration and atomic rearrangement.

#### 3.2.2 Modulation of the chemical state and coordination environment

The modulation of the chemical state and coordination environment offers a strategy to pre-design the reconstruction pathway itself.^175,176^ This strategy focuses on the dynamic evolution of the local structure of active centers during reconstruction, where the coordination number, bond length, symmetry, and valence state undergo potential-driven changes that ultimately dictate catalytic performance.^177–179^

**Fig. 12:**
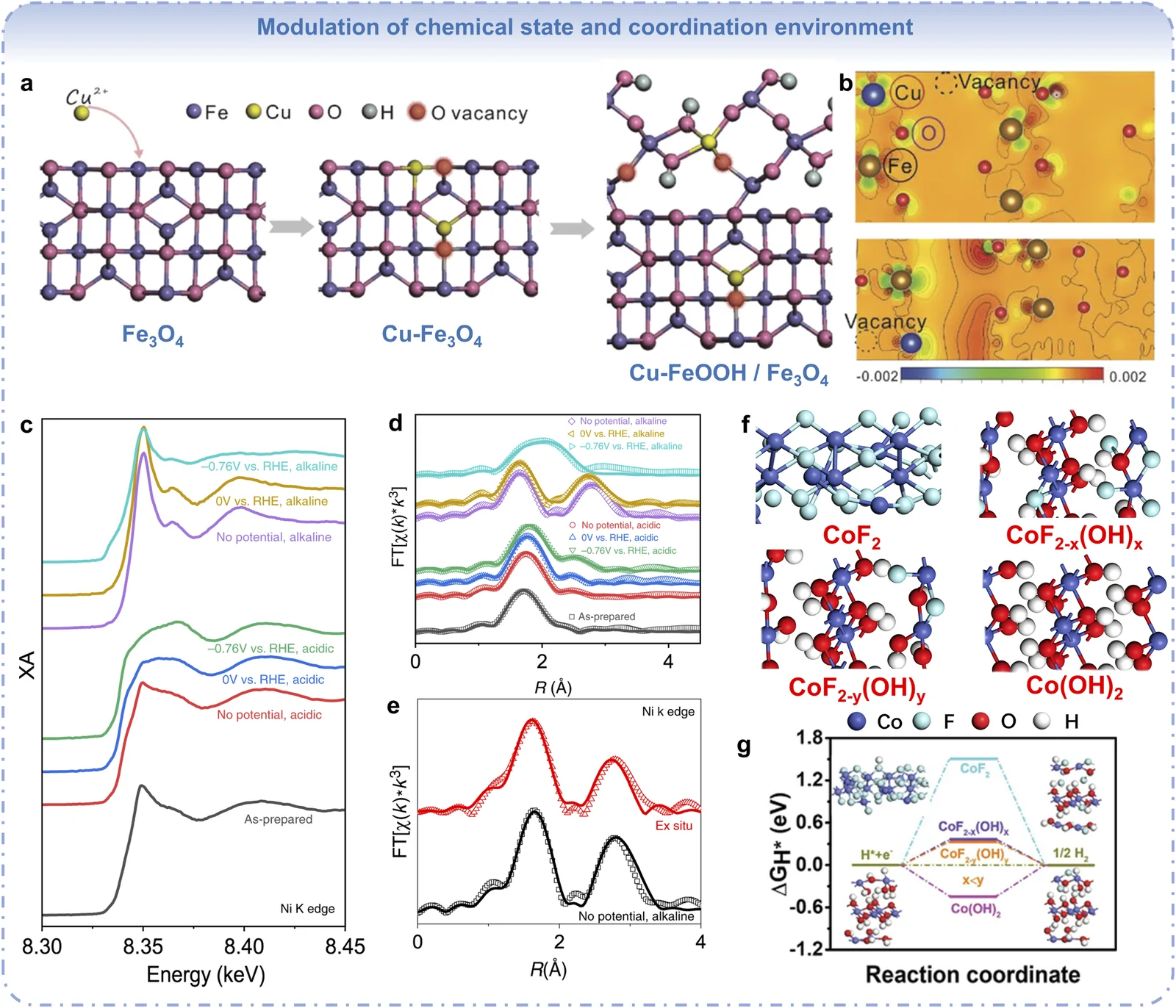
(a) Schematic diagram of the structural transformation at the atomic level. (b) 2D charge difference isosurface of a Cu-FeOOH/Fe_3_O_4_ catalyst along and vertical to the interface.^129^ Copyright 2022, Wiley-VCH. (c) Ni K-edge XANES spectra of Ni@1T-MoS_2_ under acidic conditions at different applied potentials. (d) Ni K-edge XANES spectra of Ni@1T-MoS_2_ under alkaline conditions at different applied potentials. (e) Fourier-transformed *R*-space EXAFS spectra of Ni@1T-MoS_2_ under alkaline conditions.^180^ Copyright 2020, Springer Nature. (f) Optimized structure models of CoF_2_, CoF_2−*x*_(OH)_*x*_, CoF_2−*y*_(OH)_*y*_, and β-Co(OH)_2_. (g) Calculated free energy diagram for the HER.^181^ Copyright 2021, Wiley-VCH.

This presetting logic is exemplified by Yang *et al.* in the design of Cu-FeOOH/Fe_3_O_4_ as an efficient HER catalyst.^129^ The deliberate incorporation of Cu into the Fe_3_O_4_ lattice presets a specific coordination environment around the Fe sites. At HER potentials, this Cu-modified local structure creates preferential sites for Fe leaching, and the dissolved Fe species subsequently redeposit as vacancy-rich defective FeOOH (Fig. 12a and b). The Cu dopant thus serves not merely as an electronic modifier but as a structural director that presets which sites are susceptible to leaching and how the redeposited phase nucleates. A direct demonstration of coordination-preset reconstruction comes from single-atom catalysts, where the initial coordination environment can be precisely defined. Pattengale *et al.* designed Ni single atoms anchored on 1T-MoS_2_ with well-defined Ni–S coordination.^180^ The Ni–S bonds provide the structural flexibility needed for reversible ligand exchange with hydroxyl species, while the MoS_2_ support stabilizes the isolated Ni sites against irreversible aggregation. In acid, the Ni white-line intensity changed little at applied potential, with only about 3 eV edge shift, indicating reversible reduction of Ni(ii) while maintaining its intrinsic local structure (Fig. 12c). At cathodic potentials in alkaline media, the Ni–S coordination transforms into a mixed Ni–O/S environment with the emergence of metallic Ni^0^ character (Fig. 12d and e). And upon potential removal, the system reverts to its original Ni–S state. Critically, this reversible pathway is only accessible because the initial Ni–S coordination was deliberately chosen. Ji *et al.* designed transition metal fluorides with Co–F coordination as the initial state for alkaline HER.^181^ The deliberate choice of F^−^ as the coordinating anion presets a specific reconstruction pathway, where at HER potentials the Co–F bonds undergo gradual hydrolysis, converting the coordination from Co–F to Co–OH and driving the phase evolution from amorphous to crystalline β-Co(OH)_2_ (Fig. 12f). The continuous leaching of F^−^ generates coordinatively unsaturated sites, while the newly formed Co–OH coordination provides the active motif for the HER (Fig. 12g). These studies show that by presetting the initial coordination species, one can guide the reconstruction pathway that leads to the desired active phase.

Taken together, the rational design strategies discussed in this chapter share a common thread. Whether through defect engineering or coordination regulation, they all aim to pre-design the formation of the target active phase at the atomic scale. For a side-by-side comparison, Table 2 summarizes the design strategies, reconstruction pathways, actual active species, and HER performance of representative pre-catalysts.

**Table 2:** Comparison of design strategies, reconstruction pathways, and HER performance of pre-catalysts

Design strategy type	Catalyst	Active phase	Catalyst loading (mg cm^−2^)	HER activity	Ref.
Defect/vacancy engineering	Ru-NiPS_3_ NSs	Amorphous Ru^4+^-S_2_^2−^/NiPS_3_ edge	1.5	*η*_10_ = 59 mV (1 M KOH)	182
	S-Co_3_O_4_/CC	Co-Co^δ+^S_*x*_	—	*η*_10_ = 119 mV (1 M KOH)	115
Chemical state modulation	NCMP	NiCoMn(OH)_2_	22	*η*_10_ = 100 mV (1.0 M KOH)	62
	Ni_3_S_2_/NiO	Ni_3_S_2_/NiO heterointerface	—	*η*_10_ = 95 ± 8 mV	150
	CoP|F-CFP	Amorphous Co–F	1.21	*η*_10_ = 70 Mv (0.5 M H_2_SO_4_)	183
	Cr-Ni_3_N/NF	Ni_3_N/Ni(OH)_2_	—	*η*_10_ = 72 mV (1.0 M KOH)	184
	Ni@Ni/NiO_*x*_	Ni/NiO_*x*_	12.7	*η*_10_ = 71 mV (1 M KOH)	185
	R-S-MnO_*x*_-CC	Mn_3_O_4_	2.25	*η*_10_ = 43 mV (0.5 M H_2_SO_4_)	186
	Mo_2_C-MoO_*x*_/CC	MoO_2_	1.8 ± 0.1	*η*_10_ = 60 mV (1.0 M HClO_4_)	187
	RWO	RuO_*x*_/WO_2_	—	*η*_1000_ = 317 mV (1 M KOH)	188
	Co-CoO-C/Mo_2_N/CC	Co(OH)_2_-CoO-C/Mo_2_N	4.2	*η*_10_ = 27 mV (1 M KOH)	189
	A-NiCoB/NF	CoO_*x*_/NiCoB	—	*η*_500_ = 209 mV (1 M KOH)	190
	CdNNi_3_ NS	Cd–NiO	0.5	*η*_1000_ = 235 mV (1 M KOH)	191
	Post-HER Ni_3_S_2_	Ni oxysulfide	—	*η*_10_ = 69 mV (1 M KOH)	192
Interface/heterojunction construction	CeCoFeP@	(Co,Fe)(OH)_2_·*x*PO_4_@V_2_C MXene	0.28	*η*_10_ = 91 mV; *η*_300_ = 214 mV (1 M KOH)	71
	MXene				
	Ni_0.2_Mo_0.8_N/MoO_2_@NF	Ni_0.2_Mo_0.8_N/MoO_2_ heterointerface	—	*η*_100_ = 30 mV; *η*_1000_ = 212 mV (1 M KOH, freshwater); *η*_1000_ = 217 mV (alkaline seawater)	193
	FeCoP/CF	Co(OH)_2_/CoP	—	*η*_10_ = 20 mV (1.0 M KOH)	194
	E-Ni_90_Mo_10_/CC	Ni_90_Mo_10_/Ni_*x*_Mo_*y*_O_*z*_	1.0	*η*_10_ = 56.84 mV (0.5 M H_2_SO_4_)	195
				*η*_10_ = 56.84 mV (0.5 M H_2_SO_4_)	
	Ta-doped Ni_3_S_2_	Ni oxysulfide	—	*η*_10_ = 56 mV (1 M KOH)	196
	A-NiCoPB/NF	Ni_*x*_Co_1−*x*_(OH)_2_	—	*η*_500_ = 172 mV (1 M KOH)	197
	Co(OH)_2_-ZIF@Ru/NF	Ru/Co(OH)_2_-ZIF	4.1	*η*_10_ = 22 mV	198
				*η*_100_ = 98 mV (1 M KOH)	
	NiS_2_@CC-900	Ni/Ni_3_S_2_	—	*η*_10_ = 94 mV (1 M KOH)	199

## 4 Synergistic studies by *in situ* characterization and computational simulation

The dynamic reconstruction of catalysts encompasses a succession of continuous processes including atomic migration, the breaking and reorganization of chemical bonds, and the evolution of interfacial structures.^200–202^ Conventional *ex situ* techniques can only capture the static information of the initial and final states, rendering them incapable of probing intermediate structures and transient active sites. This chapter focuses on the synergy between experiment and theory. The dynamic nature of reconstruction demands techniques capable of tracking structural evolution in real time, while the complexity of the underlying mechanisms requires theoretical frameworks to rationalize the observed phenomena.^203–205^ To address these requirements, the chapter first surveys multimodal *in situ* characterization techniques that capture dynamic evolution across electronic, structural, and surface chemical levels. It then examines multi-scale computational methods, ranging from density functional theory to machine-learned force fields, that provide atomistic insights into reconstruction pathways. The discussion culminates in the cross-validation between experiment and computation, which enables the construction of a data-driven dynamic structure–activity relationship model and lays a methodological foundation for the rational design of catalysts.

### 4.1 *In situ* characterization techniques

Establishing the HER catalytically active phase requires evidence obtained under operating conditions, rather than conclusions drawn solely from post-reaction characterization or DFT calculations. The *in situ* and *operando* characterization data are recognized as the primary evidence for tracking structural evolution of HER pre-catalysts under reaction conditions. These are clearly distinguished from *ex situ* measurements, such as XPS, XRD and TEM, which are regarded as complementary indicators of the recovered state rather than direct evidence of the working phase and are subject to structural relaxation or surface oxidation upon removal from potential and exposure to air. The complexity of reconstruction lies in the fact that it simultaneously involves changes in electronic structure parameters such as the oxidation state and coordination environment, as well as atomic-scale structural evolution including lattice arrangement and phase boundary migration.^206,207^ A single technique can often provide only a glimpse of one facet, making multimodal combination an inevitable choice. The complementarity of multiple technologies should lie in the detection of large-scale atomic and electronic structures, as well as the identification of surface substances and reaction intermediates.

**Fig. 13:**
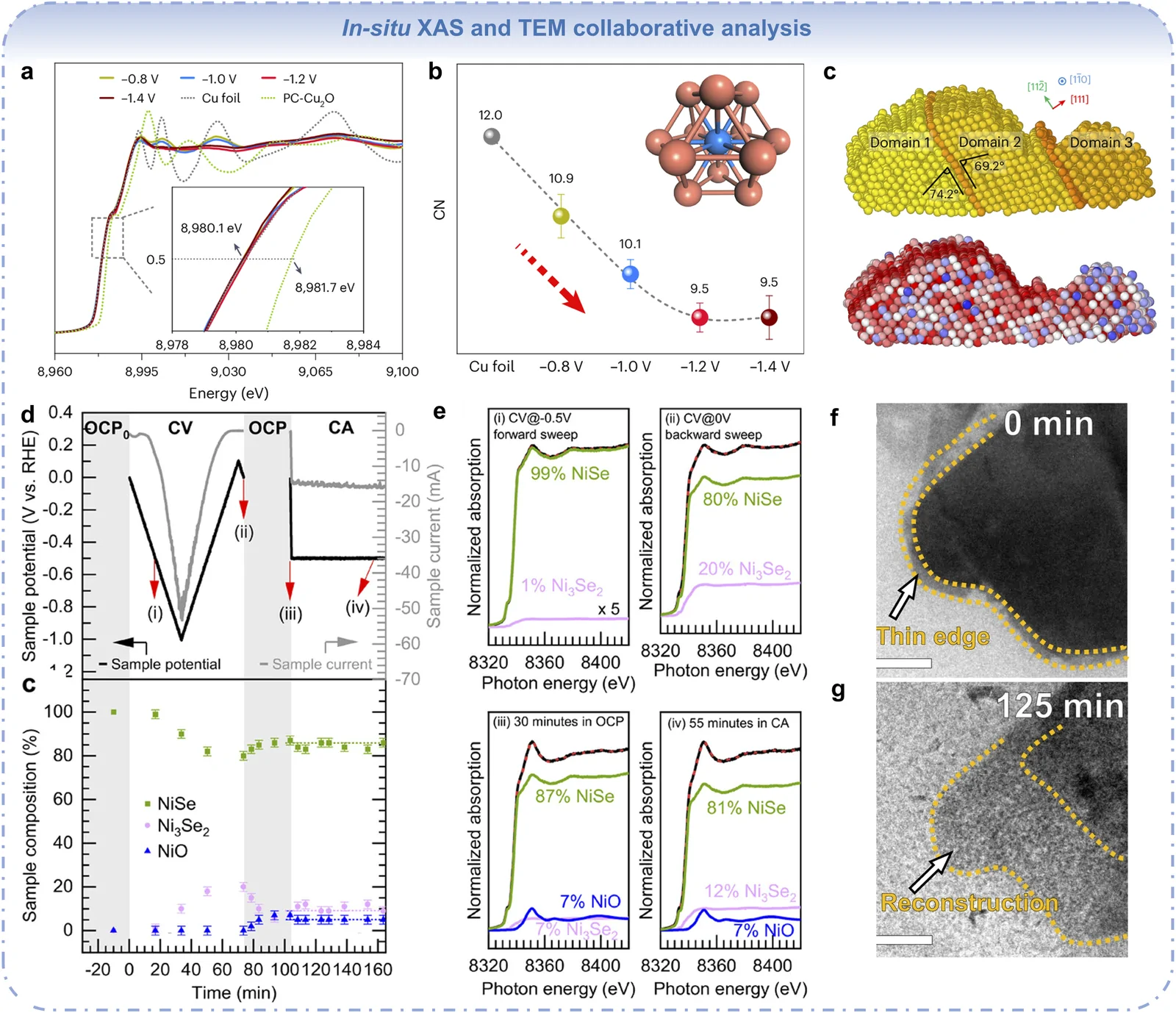
(a) *In situ* XANES spectra of PC-Cu_2_O and DNTs-Cu obtained at different electroreduction potentials. (b) Radial distribution functions of DNTs-Cu catalysts at different electroreduction potentials and Cu foil. (c) 3D atomic model of a DNTs-Cu particle showing domains separated by twin boundaries and a dislocation.^164^ Copyright 2025, Springer Nature. (d) *Operando* Ni K-edge XAS spectra of NiSe and their linear combination of NiSe, Ni_3_Se_2_, and NiO corresponding to different electrochemical testing stages. (e) Phase compositions at different stages of the *in situ* electrochemical testing process.^105^ Copyright 2026, Wiley-VCH. (f) *In situ* liquid TEM image of Ru-NiPS_3_ NSs before the chronopotentiometry test. (g) *In situ* liquid TEM image of Ru-NiPS_3_ NSs after the chronopotentiometry test.^182^ Copyright 2023, Springer Nature.

*In situ* X-ray absorption spectroscopy (XAS) and *in situ* transmission electron microscopy (TEM) respectively provide statistically averaged global information and locally intuitive structural images, forming a complementary cross-scale analytical capability. Within XAS, X-ray absorption near-edge structure (XANES) is highly sensitive to the oxidation state and local symmetry of the central atom, making it suitable for tracking the continuous evolution of metal valence states during reconstruction. Extended X-ray absorption fine structure (EXAFS), through the interference effect of photoelectron backscattering, provides quantitative parameters such as the coordination number, bond length, and disorder degree. Capitalizing on these techniques, Li *et al.* combined *in situ* XAS to track the atomic structure evolution during the electroreduction of polycrystalline Cu_2_O to Cu.^164^ XANES confirmed the reduction from Cu_2_O to metallic Cu through the absorption edge shift from 8981.7 eV to 8980.1 eV (Fig. 13a). EXAFS analysis revealed that the Cu–Cu bond length increased to 2.255 Å and the average coordination number decreased to 9.5 as the reduction potential was lowered (Fig. 13b). As shown in the three-dimensional atomic reconstruction (Fig. 13c), Cu atoms with coordination numbers ≤5 and tensile strain ≥6% were identified as highly active sites, providing direct atomic-scale evidence for the low coordination-strain synergy mechanism. Ding *et al.*, using a NiSe single crystal as a model system, tracked its alkaline HER reconstruction *via operando* XAS (Fig. 13d).^105^ Linear combination fitting of the near-edge spectra revealed that pristine NiSe initially transformed into Ni_3_Se_2_ during cyclic voltammetry, as confirmed by the appearance of a Ni–Se scattering path at 2.36 Å. Upon further polarization at cathodic potentials, a Ni–O scattering path emerged, indicating the formation of NiO species on the surface. The sequential evolution from NiSe to Ni_3_Se_2_ and finally to a Ni_3_Se_2_/NiO heterojunction provides direct spectroscopic evidence for the phase transformation pathway (Fig. 13e). Post-HER XRD confirms that NiSe forms a comb-like Ni_3_Se_2_/NiO heterojunction layer on the surface, proving that the observed bonding changes correspond to the phase reconstruction rather than reversible surface adsorption.

In contrast to the statistical averaging of XAS, *in situ* TEM directly visualizes the dynamic evolution of the lattice structure. Lattice fringes in high-resolution mode can resolve the nucleation and growth of new phases and the trajectory of dislocation movement, while geometric phase analysis can convert lattice displacements into a two-dimensional strain field map.^208,209^ Fu *et al.*, using *in situ* liquid-phase electrochemical TEM, observe the edge amorphization evolution of Ru-doped NiPS_3_ nanosheets during alkaline HER.^182^ With prolonged reaction time, the thickness of the amorphous edge layer increased to approximately 7.5 nm before stabilizing, while the crystalline core remained unchanged (Fig. 13f and g), clearly revealing the evolutionary pathway of a surface-amorphized and bulk-crystalline core–shell structure. In summary, XAS indicates the statistical trend of structural change, while TEM reveals the specific location and manner in which the change occurs.

**Fig. 14:**
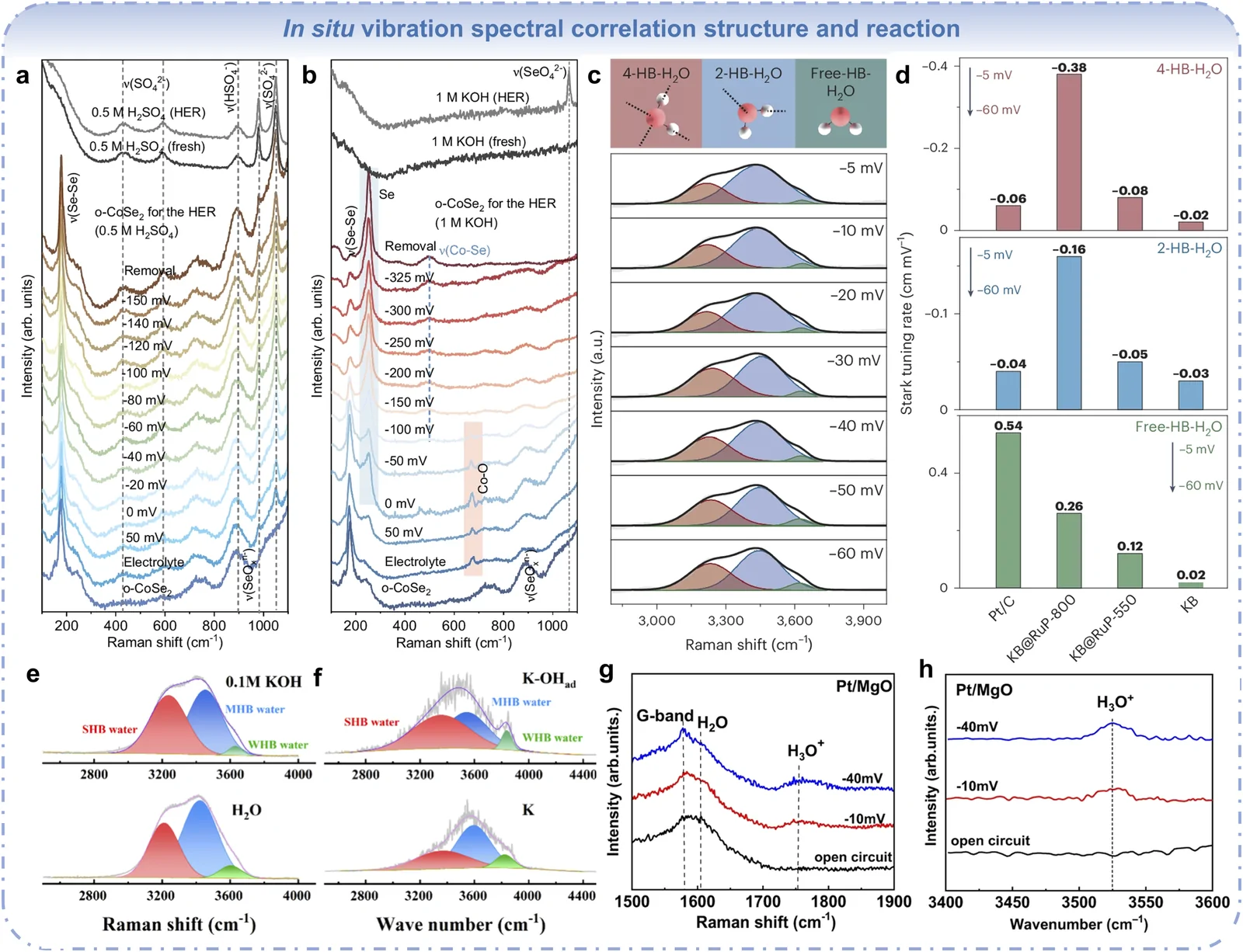
(a) *Operando* Raman spectra of *o*-CoSe_2_ during acidic HER. (b) *Operando* Raman spectra of *o*-CoSe_2_ during alkaline HER.^214^ Copyright 2025, Wiley-VCH. (c) *In situ* Raman spectra of KB@RuP-800 at different potentials with Gaussian deconvolution. (d) Proportion of three peaks of interfacial water on KB@RuP-800 from 0.00 to −0.20 V.^215^ Copyright 2025, Springer Nature. (e) Gaussian deconvolution of the O–H stretching band from surface-enhanced Raman spectra of the Pt electrode in 0.1 M KOH solution and pure water. (f) Gaussian deconvolution of VDOS for the O–H stretching mode before and after OHad adsorption, with the same three-component assignment as in (e).^216^ Copyright 2026, American Chemical Society. (g) *Operando* Raman spectra of Pt/MgO in 1 M KOH. (h) *Operando* SR-FTIR spectra of Pt/MgO in 1 M KOH.^219^ Copyright 2022, Springer Nature.

While XAS and TEM focus on the structural evolution of the catalyst itself, they cannot directly observe the chemical reactions occurring on the surface. Bridging the cognitive gap between the catalyst structure and catalytic reaction requires spectroscopic techniques sensitive to surface species, capable of tracking the adsorption, transformation, and desorption behavior of reaction intermediates in real time. *In situ* Raman spectroscopy, based on the inelastic scattering of incident light coupled with molecular vibrations, possesses high sensitivity toward the vibrational modes of metal–oxygen bonds, metal–hydrogen bonds, and bonds between chalcogenide elements.^210–213^ A comprehensive demonstration of this capability is provided by Zhao *et al.*, who employed *operando* Raman spectroscopy combined with X-ray absorption spectroscopy to track the pH-dependent reconstruction pathways of CoSe_2_.^214^ By comparing the behavior of *o*-CoSe_2_ in both acidic and alkaline media, the *operando* Raman spectra captured distinct vibrational fingerprints of transient surface species evolving with the applied potential. Under acidic conditions, the intrinsic Se–Se vibrational modes remained unchanged (Fig. 14a), whereas in alkaline media, a distinctly different spectral evolution was observed. The Se–Se and SeO_*x*_^*n*−^ signals both exhibited progressive weakening upon cathodic polarization. A new Co–O vibration emerged at above −100 mV *vs.* RHE; at more negative potentials, this Co–O feature disappeared and was replaced by new Co–Se vibrations. After potential removal, a pronounced SeO_4_^2−^ signal was detected in the electrolyte, confirming that the surface oxidized species had undergone further restructuring into active Co species during the alkaline HER (Fig. 14b). The *in situ* Raman spectra thus captured the real-time evolution of surface species under different conditions, revealing that *o*-CoSe_2_ undergoes only partial surface corrosion in acid but a complex restructuring pathway in alkali, involving initial hydroxylation followed by reduction to metallic Co species. Li *et al.*, through *in situ* Raman spectroscopy, analyzed the interfacial water configuration on the surface of RuO_2_@RuP_2_ core–shell catalysts.^215^ As the cathodic potential increased, the proportion of 4-coordinated hydrogen-bonded water rose while that of 2-coordinated hydrogen-bonded water decreased (Fig. 14c and d), indicating that the catalyst surface actively reconstructed the interfacial water network to facilitate proton transfer. Complementing these studies, Lian *et al.* employed *operando* surface-enhanced Raman spectroscopy (SERS) to probe the restructuring of the electric double layer water network during alkaline hydrogen evolution.^216^ After OHad adsorption, the SHB component significantly increased, while the WHB component decreased significantly (Fig. 14e). The vibrational density of states calculated based on AIMD trajectories also reproduces the same spectral trend (Fig. 14f).

By comparison, *in situ* synchrotron-radiation Fourier-transform infrared spectroscopy (SR-FTIR) exploits the plasmon resonance of metal surfaces to enhance the infrared signals of adsorbed molecules, offering superior detection capability for adsorbed water configurations, hydroxyl species, and hydrogen-bond networks, making it particularly suitable for probing the dynamic reconstruction of the electrical double layer under operating conditions.^217,218^ Tan *et al.* combined *in situ* Raman spectroscopy (Fig. 14g) with SR-FTIR (Fig. 14h) to monitor the interfacial evolution on Pt/MgO during alkaline HER.^219^ Under cathodic polarization, the interfacial water network undergoes a structural reorganization, with the H_2_O signal progressively weakening while a new H_3_O^+^ feature emerged at 3525 cm^−1^ and intensified with increasing potential. This H_3_O^+^ signal was reversible, appearing only at HER potentials and disappearing upon potential removal, indicating a potential-dependent reorganization of the interfacial hydration environment driven by the oxide support.

Although electrochemical *in situ* characterization techniques are some of the advantageous tools for obtaining the structure, composition and surface changes of pre-catalysts for the HER, there are still limitations in their application. Each tool has its own advantages and disadvantages in terms of time and spatial resolution, sensitivity to local and overall structures, and the ability to capture the dynamic characteristics of the reconstructed surface. *In situ* TEM offers real-space imaging of morphological evolution but suffers from electron-beam effects, high-vacuum conditions that deviate from actual electrochemical environments, and limited statistical representativeness; *in situ* Raman spectroscopy is surface-sensitive but has limited probe depth for subsurface or amorphous components; *in situ* SR-FTIR provides valuable information on interfacial species and adsorbed intermediates, yet its signal is often complicated by strong water absorption and spectral overlap of multiple surface species, and its spatial resolution remains limited. Also, it is necessary to distinguish between structural changes observed by *in situ* techniques and the causal relationship associated with the enhancement of HER activity. Even with synchronous *in situ* spectroscopy and electrochemical responses providing support, the correlation itself does not prove the final conclusion.

### 4.2 Mechanistic elucidation and pathway prediction: multi-scale computational methods

Computational simulation provides complementary tools for probing the thermodynamic tendency, atomic-scale mechanisms, and possible pathways of reconstruction.^220,221^ It transcends the limitations of experimental spatiotemporal scales, revealing at the electronic level why defects or interfaces become initiation sites for reconstruction, clarifying how reconstruction modulates the electronic structure, and establishing quantifiable structure–activity relationships.

#### 4.2.1 Thermodynamic stability analysis and kinetic pathway simulation

Density functional theory (DFT) provides the foundation for understanding why reconstruction occurs and where it initiates.^222^ DFT-based stability calculations can construct potential-composition phase diagrams to forecast thermodynamically favorable reconstruction products under given operating conditions.^223^ The d-band center serves as a key descriptor linking the electronic structure to reconstruction propensity, as its position relative to the Fermi level dictates both the adsorption strength of intermediates and the stability of metal-anion bonds.^224–226^ Crystal orbital Hamilton population (COHP) analysis further reveals bond weakening and reorganization during reconstruction by quantifying bond covalency and strength.^227–230^ Zhan *et al.* systematically compared the electronic structures of perfect MoS_2_, S-terminated vacancies, and Mo-terminated vacancies using DFT, demonstrating its power in resolving reconstruction mechanisms.^116^ Electron localization function and Bader charge analyses showed that in S-terminated vacancies, residual electrons flow to the surrounding S atoms, whereas in Mo-terminated vacancies, they flow to the exposed Mo atoms. COHP analysis further establishes a quantitative correlation between the ICOHP value and adsorption strength, elucidating the electronic origin of vacancy-mediated HER activity modulation. While DFT remains a valuable tool for comparing the relative stability or reactivity of proposed structural models, it still has intrinsic limitations, including idealized surface terminations, neglect of solvation effects and applied electrode potential in most models, and the difficulty of adequately sampling the configurational space of disordered or amorphous reconstructed layers. So, individual DFT computational results should be used to generate testable hypotheses that are assessed against experimental observations, rather than as standalone proof of the active phase.

**Fig. 15:**
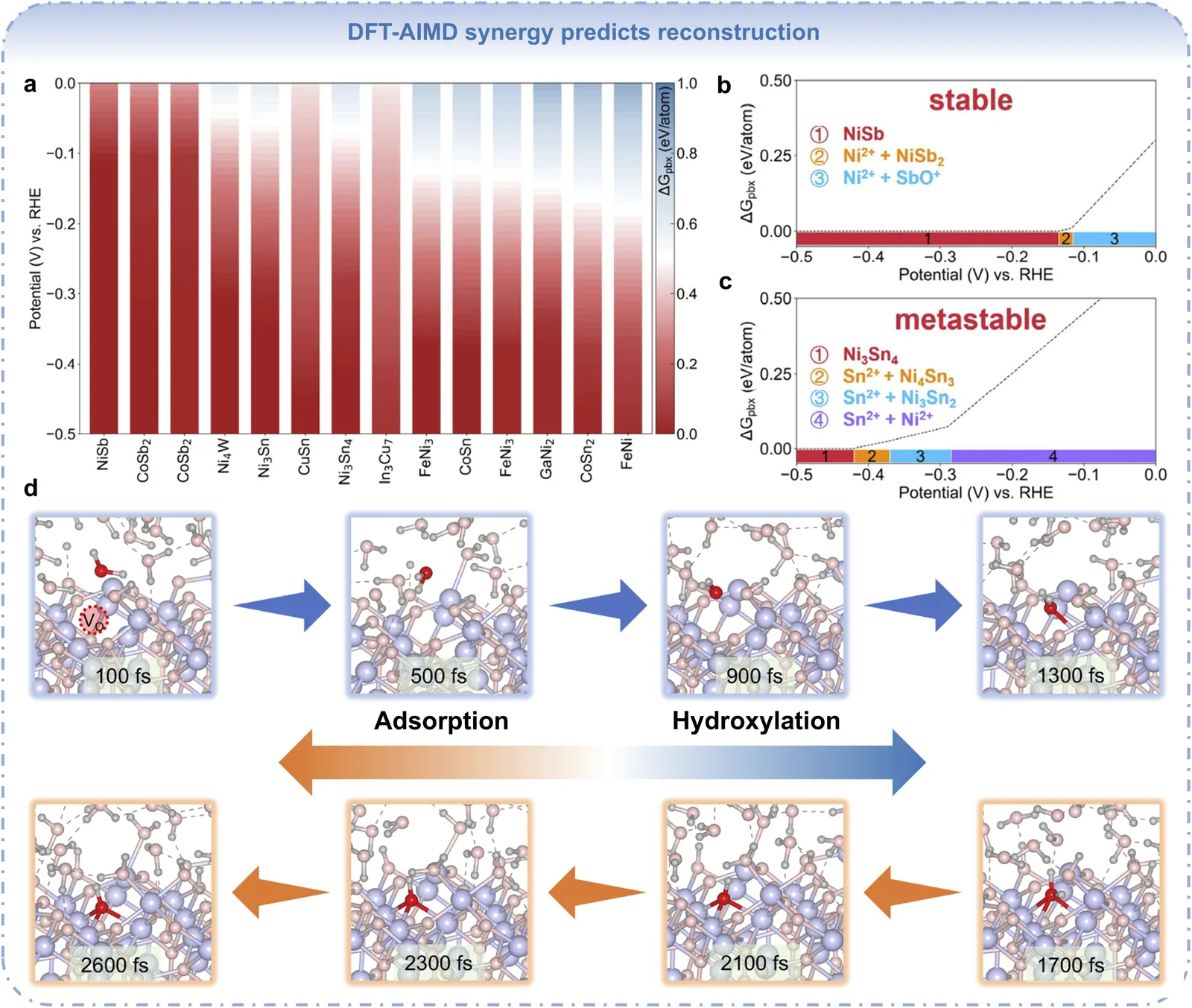
(a) The 14 acid-stable PGM-free binary alloy candidates identified through the stability–activity-price screening framework. The Δ*G*_pb*x*_ of the NiSb alloy (b) and Ni_3_SN_4_ alloy (c).^234^ Copyright 2025, American Chemical Society. (d) AIMD simulations for the processes of water molecule adsorption and surface hydroxylation induced by oxygen vacancies on Co_3_O_4_.^237^ Copyright 2026, American Chemical Society.

Beyond electronic structure analysis, DFT-based stability calculations can be used to construct Pourbaix diagrams to forecast thermodynamically favorable phases under given pH and potential conditions.^231–233^ These diagrams enable systematic screening of materials for electrochemical stability before experimental synthesis. The predictive power of Pourbaix analysis for HER catalyst stability is demonstrated by Cai *et al.* with a high-throughput screening study on 185 binary alloys for acidic HER.^234^ The Pourbaix decomposition free energy (Δ*G*_pb*x*_) was used as a stability descriptor to screen candidates across potential-pH space. With a stability criterion of Δ*G*_pb*x*_ ≤ 1.0 eV per atom under HER-relevant conditions, combined with cost and activity thresholds, 14 acid-stable PGM-free candidates were identified (Fig. 15a). Among them, NiSb emerged as the most promising, with a maximum Δ*G*_pb*x*_ of only 0.30 eV per atom (Fig. 15b). The theoretical predictions were experimentally validated by synthesizing the NiSb catalyst, confirming the reliability of Pourbaix-based stability screening. In addition, the Pourbaix diagram can also provide detailed information on phase transformation and stable regions of the HER catalyst under varying electrochemical conditions (Fig. 15c). Zhu *et al.* employed surface Pourbaix diagram calculations to map the potential-dependent phase evolution of Co_2_Mo_3_O_8_.^123^ For the two-dimensional surface Pourbaix diagram of the Co end face (001), the stability of each surface species in the potential-pH space was quantified. At oxidative potentials exceeding 0.3 V *vs.* SHE, the Co_2_Mo_3_O_8_ (001) surface is covered by O and prone to CoO_*x*_ formation. As the potential shifts to the reduction region (−0.39 V *vs.* RHE), CoO_*x*_ transforms into Co(OH)_2_. At more negative potentials, the surface becomes H-covered, a state that protects the structure from severe etching. These theoretical predictions are in good agreement with experimental observations. Guided by this understanding, the catalyst exhibiting the most extensive Co(OH)_2_/Co_2_Mo_3_O_8_ interface also delivered the highest HER activity.

While Pourbaix analysis predicts the thermodynamically stable phase under given conditions, the experimentally observed reconstructed layer is often kinetically trapped or dynamically maintained rather than a global minimum. Factors including potential history, kinetic barriers, mass transport, local pH gradients, and defect density can all cause deviations from equilibrium predictions. Reconstruction frequently terminates at metastable structures such as amorphous layers, heterointerfaces, or defect-rich phases. These may either evolve further under prolonged polarization or persist as the steady-state catalytic structure if the activation barriers for further transformation are not overcome. The steady-state structure thus represents the functional state under operating conditions, rather than necessarily being the thermodynamically preferred phase or the initial kinetic product.

Also, Pourbaix diagrams are equilibrium constructs. They assume bulk-phase equilibrium with the electrolyte and ideal crystallinity and therefore predict the thermodynamically most stable phase at a given potential and pH. They do not contain any information about kinetic barriers or transformation pathways. However, the actual reconstructed active phase is often kinetically arrested. In HER catalysts, reconstruction frequently leads to heterointerfaces, amorphous layers, or defect-rich phases that are not the global thermodynamic ground state. These structures persist because the activation barriers for further transformation are not overcome at the applied overpotential. Also, real reconstructed catalysts deviate strongly from ideal bulk assumptions. Lattice disorder, non-uniform strain fields, nanoscale dimensions, and high defect densities are intrinsic to practical systems, and these features lie outside the scope of equilibrium phase diagrams. Several HER reconstruction studies have demonstrated such deviations between thermodynamic prediction and experimentally observed working states. For example, in HER electrocatalysis, amorphous MoS_2_ represents a metastable structure that cannot be simply described by conventional equilibrium phase diagrams. Although crystalline 2H-MoS_2_ is thermodynamically stable, *operando* studies demonstrated that amorphous MoS_2_ can retain distinct Mo–Mo bonding characteristics and sustained HER activity, whereas metastable 1T-MoS_2_ gradually evolves under HER conditions.^235^ Similarly, cobalt phosphide catalysts have been reported to undergo local reconstruction into amorphous Co(OH)_2_-containing structures during HER-related operation. Such reconstructed configurations arise from nanoscale rearrangement and local coordination changes rather than direct transformation into a bulk equilibrium phase predicted by Pourbaix analysis.^236^ So, Pourbaix analysis is used only as a thermodynamic boundary marker. It serves to define the feasible phase space for reconstruction, but it does not provide a deterministic description of the final active structure.

While DFT provides thermodynamic information about possible reconstruction products, the kinetic pathway and time scale of reconstruction require dynamic simulation methods. *Ab initio* molecular dynamics (AIMD) calculates atomic forces *via* DFT at each time step and solves Newton's equations of motion, enabling direct simulation of atomic trajectories in explicitly modeled interfacial environments. This method can capture transient processes that are experimentally difficult to observe, including how lattice atoms detach from the surface, how chemical bonds break and reorganize, and how water molecules participate in reconstruction. The capability of AIMD to capture dynamic surface processes is demonstrated by Wang *et al.* in their study of oxygen vacancy evolution on Co_3_O_4_ during alkaline HER.^237^ AIMD simulations revealed that oxygen vacancies are not inert sites but are dynamically consumed under cathodic polarization, promoting extensive surface hydroxylation (Fig. 15d). This vacancy-driven hydroxylation, in turn, modulates the distribution of interfacial water molecules and enhances the connectivity of the hydrogen-bond network. The simulated structural evolution was corroborated by *operando* Raman spectroscopy and X-ray absorption spectroscopy, confirming that the hydroxylated surface facilitates water dissociation and shifts the reaction pathway from Volmer–Heyrovsky to Volmer–Tafel. This work illustrates how AIMD can provide atomistic insight into vacancy consumption, surface hydroxylation, and interfacial-water evolution associated with HER reconstruction. It should be noted that conventional AIMD simulations are generally limited by computational cost and simulation timescale, and they do not directly reproduce the dynamic electrochemical environment with controlled electrode potentials. The standard AIMD simulations are typically performed at constant temperature and volume without explicit control of electrode potential, and thus the structural evolution observed in such simulations does not automatically represent the behavior under electrochemical polarization. The absence of solvated counterions, explicit potential control, or an applied electric field means that AIMD can capture thermally driven atomic rearrangements but may not faithfully reproduce the potential-driven reconstruction processes that occur at the electrode–electrolyte interface under operating conditions. It is also noted that AIMD is limited to nanosecond timescales and system sizes on the order of hundreds of atoms, which restricts its ability to sample slow kinetic processes such as long-range ion diffusion or phase nucleation. The AIMD results are now presented as capturing the intrinsic structural lability and local coordination preferences of the precursor under thermal activation, rather than as a direct simulation of potential-driven reconstruction. Therefore, AIMD should mainly be regarded as a complementary tool for mechanistic understanding rather than a fully predictive approach for HER reconstruction.

These computational approaches address the reconstruction problem from complementary angles. Electronic structure analysis reveals why certain sites become initiation points for reconstruction. Pourbaix diagrams establish the thermodynamic boundaries within which reconstruction can occur. AIMD captures the kinetic pathways through which reconstruction proceeds. The integration of these methods encourages the design of pre-catalysts with targeted reconstruction pathways, where the initial structure is designed to contain the thermodynamic and kinetic information needed to guide its evolution toward the desired active phase under operating conditions.

#### 4.2.2 Bridging the scale gap with machine-learned force fields

Bridging the scale gap between microscopic mechanisms and macroscopic performance hinges on the development of simulation methods with larger spatiotemporal scope. Machine-learned force fields (MLFFs) are trained on large datasets of atomic configurations and energies generated from DFT calculations, using neural networks or high-dimensional polynomials to represent interatomic potentials. Once trained, MLFFs achieve near-DFT accuracy with several orders of magnitude higher computational efficiency, enabling simulations at nanometer scales and nanosecond to microsecond time scales. This capability is particularly valuable for studying complex interfacial reconstruction, long-term evolution of defect networks, and multi-grain interactions. Advances in active learning cycles have further enhanced the construction efficiency and generalizability of MLFFs, enabling iterative refinement of force fields with minimal human intervention. In the context of HER pre-catalyst reconstruction, MLFFs serve two complementary functions. The first is to extend the timescale of atomistic simulations beyond the picosecond limit of AIMD, enabling observation of structural evolution from initiation to completion. The second is to accelerate the exploration of composition-structure–activity relationships that underpin rational pre-catalyst design.

**Fig. 16:**
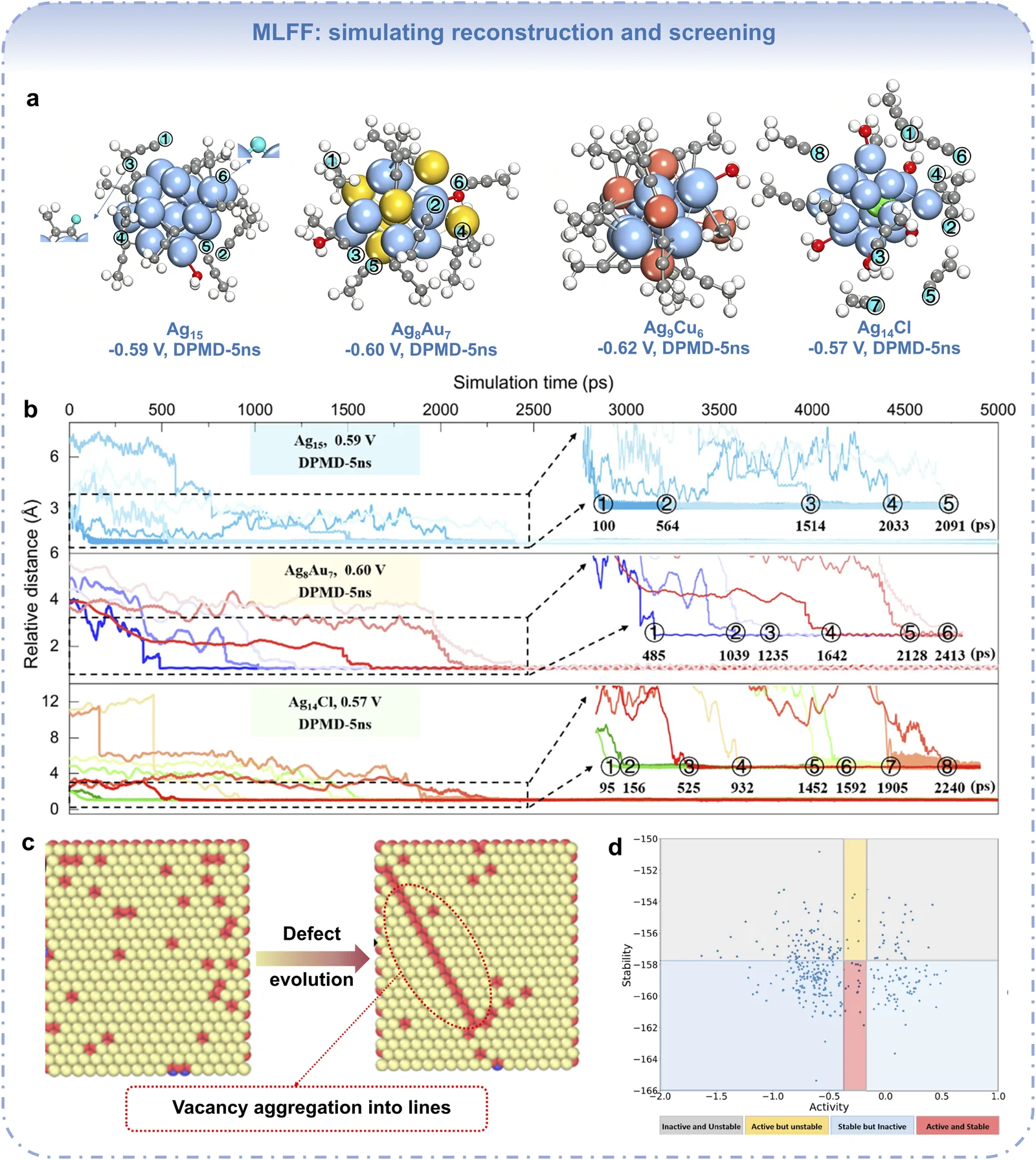
(a) DPMD snapshots of Ag_15_, Ag_8_Au_7_, Ag_9_Cu_6_, and Ag_14_Cl nanoclusters. (b) Dynamic changes of H moving from the water layer to the terminal C atom over time during the DPMD simulation.^238^ Copyright 2025, American Chemical Society. (c) Evolution of Mo atoms proximal to line defects at 900 K.^239^ Copyright 2018, American Chemical Society. (d) High-throughput screening results of the surface prediction model for amorphous Ni_2_P.^240^ Copyright 2020, American Chemical Society.

MLFFs have proven particularly valuable in capturing structural evolution phenomena that span timescales beyond the reach of AIMD. A case in point is the work of Tang and colleagues on alkynyl-protected silver nanoclusters and their doped variants.^238^ The central question was whether these atomically precise clusters retain their structural integrity under electrochemical conditions or undergo dynamic reconstruction. AIMD simulations revealed the early stages of this process: the octahedral core began to disorder and surface ligands partially detached. However, the picosecond timescale accessible to AIMD was insufficient to determine whether these structural changes represented a transient response or a completed reconstruction. By extending the simulation time to nanoseconds through MLFF-driven deep potential molecular dynamics, the study captured the full trajectory of reconstruction. The DPMD simulations (Fig. 16a) reveal that the final number of completely desorbed ligands depends critically on the dopant type and time-resolved analysis (Fig. 16b) and further demonstrated that proton transfer from the water layer to the terminal alkyne carbon occurred at distinctly different timescales and that simulations beyond 2000 ps are essential to reach the true steady state. This work demonstrates that MLFFs can bridge the critical timescale gap in reconstruction studies, enabling the observation of structural evolution from initiation to completion without sacrificing first-principles accuracy. This timescale advantage also applies to capturing defect-mediated transformations in two-dimensional materials. Patra *et al.* published a study on monolayer MoS_2_ combined genetic algorithms with MLFF-driven molecular dynamics to investigate the extended structure of point defects and their dynamic evolution.^239^ The challenge was that both experiments and conventional MD were limited in studying defect evolution due to insufficient spatiotemporal resolution or time scale. The MLFF provides the critical efficiency gain, enabling near-DFT-accuracy MD simulations over extended time scales. The simulations revealed that sulfur vacancies organize into extended line defects, which locally trigger the semiconducting 2H to metallic 1T phase transition (Fig. 16c). This study provides a useful methodological example of how MLFFs can access defect evolution over spatial and temporal scales that are difficult to reach with conventional first-principles simulations. Note that these MLFF examples are intended to offer mechanistic insights into the structural lability and possible transformation pathways of the precursor, but they do not constitute a direct simulation of HER potential-driven reconstruction.

Beyond simulating dynamic processes, MLFF-accelerated frameworks have also been developed for high-throughput exploration of HER catalysts. Zhang and colleagues trained ML models on DFT-calculated chemisorption energies to screen active sites on amorphous catalytic surfaces among 600 surface configurations (Fig. 16d).^240^ Although this work concerns static activity prediction rather than reconstruction, it illustrates how ML-assisted screening can efficiently map composition–structure–activity relationships and may provide methodological guidance for future reconstruction studies. A complementary descriptor-based approach is demonstrated by Wang and colleagues for 2D MXene HER electrocatalysts.^241^ By combining DFT calculations with a mechanism-guided descriptor, they established composition-activity trends that enable rational catalyst design without exhaustive computational screening. While this work did not directly simulate reconstruction, the descriptor-based framework illustrates how ML-accelerated approaches can map the relationship between material composition, electronic structure, and catalytic performance.

The expanding role of MLFFs and related ML methods in HER electrocatalysis research can be viewed. In the specific context of pre-catalyst reconstruction, MLFFs offer a path toward simulating the complete trajectory of structural evolution under operating conditions, from the initial activation of the precursor through intermediate metastable states to the final active phase. This capability, combined with ML-accelerated screening of candidate compositions,^242^ provides a computational foundation for the rational design of pre-catalysts with tailored reconstruction pathways. For HER pre-catalyst reconstruction, MLFFs therefore represent a promising route for extending atomistic simulations to longer timescales and larger systems. Despite these advantages, the predictive capability of MLFF simulations strongly depends on the quality and diversity of the training dataset. Atomic configurations or chemical environments beyond the sampled training space may introduce uncertainties. This limitation is particularly relevant for HER reconstruction, where dynamic interfaces involve potential-dependent charge redistribution, solvent interactions, and continuously evolving surface structures.

## 5 Summary and outlook

This review has systematically surveyed the research progress in dynamic reconstruction of HER pre-catalysts, spanning reconstruction pathways and mechanisms, driving forces and rational design strategies, and the synergistic methodology of *in situ* characterization and computational simulation. Three overarching principles emerge from this comprehensive survey. The first is chemically driven interfacial optimization. Regardless of the specific leaching or dealloying pathway, HER pre-catalyst reconstruction is directed toward thermodynamically more favorable or kinetically trapped states under reductive conditions, generating functionally specialized dynamic interfaces. The second is the reconstruction pathways, in which defect engineering, chemical-state modulation, and interfacial confinement are explored as means of biasing reconstruction toward desired working states. The third is closed-loop synergy between characterization and computation: breakthroughs in mechanistic understanding arise from the deep interplay of *in situ* XAS, TEM, vibrational spectroscopy, DFT, AIMD, and MLFF, where experimental constraints and computed energetics drive each other forward. Despite these advances, a tangible gap remains between academic understanding and industrial practice. Key challenges include reconstruction stability of non-noble-metal pre-catalysts at practical current densities, accurate pathway prediction in complex electrolytes, atomistic dissection of cooperative interfaces, and the construction of a data-driven design paradigm.

Significant progress has been made in recent years in understanding the reconstruction behavior of HER pre-catalysts and translating these insights into rational design strategies. However, several fundamental issues and critical bottlenecks remain for practical industrial applications, necessitating further advancement in future research. A major challenge for reconstruction-guided catalyst design is the absence of a universal descriptor capable of quantitatively evaluating reconstruction propensity. Accurate prospective prediction in complex electrochemical systems remains challenging and is rarely achieved in the current reports. The structural and electronic parameters discussed, including d-band position, defect density, bond covalency, *etc.*, should be regarded as complementary descriptors rather than independent criteria that determine reconstruction behavior. Future development of reconstruction descriptors may require the integration of thermodynamic, kinetic, and electronic information. Thermodynamic descriptors, such as bulk/surface Pourbaix stability and vacancy formation energies, may provide insights into whether a precursor tends to undergo reconstruction or dissolution. Kinetic descriptors, including atomic diffusion barriers and structural flexibility, may help evaluate whether a reconstructed structure is accessible within practical operating timescales. Electronic descriptors, such as d-band characteristics and metal–ligand interactions, may correlate reconstructed structures with their catalytic properties. Mechanistically, the field must shift from static comparisons of pre- and post-reconstruction structures toward real-time tracking of transient bond breaking and formation. At the design level, multi-dimensional synergistic strategies integrating electronic modulation, interfacial confinement, and defect pre-design should be developed to move from passive observation to deliberate control of reconstruction pathways. Recent computational studies have provided preliminary attempts toward predictive reconstruction analysis. However, these approaches remain system-dependent, and a generally applicable descriptor framework for predicting HER reconstruction has not yet been established. At the application level, cross-scale structure–activity relationships spanning from nanoscale catalysts to macroscopic electrodes need to be established, with particular attention to stability degradation mechanisms of non-noble-metal systems at high current densities and under prolonged operation. Looking further ahead, precise mastery of dynamic reconstruction may give rise to self-adaptive catalytic materials, where synthesis, activation, and operation are seamlessly integrated. With continued progress in mechanistic understanding and the deepening synergy among *in situ* characterization, computation, and data-driven design, green hydrogen technology will play an ever-critical role in the global energy transition.

## Author contributions

Xin Zhang: conceptualization, methodology, writing. Jinrui Hu: methodology, data curation. Xinyi Yao: visualization, data curation. Wei Yan: methodology, data curation. Linda Zhang: methodology, visualization. Yawen Tang: conceptualization, methodology. Meng Li: supervision, conceptualization, writing-review & editing. Hao Li: supervision, writing-review & editing, funding acquisition. Gengtao Fu: supervision, writing-review & editing, funding acquisition.

## Conflicts of interest

The authors declare no conflict of interest.

## Data Availability

No new data were generated for this review article.
